# Detailed phenotypic and functional characterization of CMV-associated adaptive NK cells in rhesus macaques

**DOI:** 10.3389/fimmu.2022.1028788

**Published:** 2022-11-25

**Authors:** Mohammad Zahidul Hasan, Charlotte Höltermann, Beatrix Petersen, Annette Schrod, Kerstin Mätz-Rensing, Artur Kaul, Gabriela Salinas, Ralf Dressel, Lutz Walter

**Affiliations:** ^1^Primate Genetics Laboratory, German Primate Center, Leibniz Institute for Primate Research, Göttingen, Germany; ^2^PhD program Molecular Biology of Cells, GGNB, Georg August University, Göttingen, Germany; ^3^Animal Husbandry, German Primate Center, Leibniz Institute for Primate Research, Göttingen, Germany; ^4^Pathology Unit, German Primate Center, Leibniz Institute for Primate Research, Göttingen, Germany; ^5^Infection Biology Unit, German Primate Center, Leibniz Institute for Primate Research, Göttingen, Germany; ^6^NGS Core Unit for Integrative Genomics, Institute of Human Genetics, University Medical Center Göttingen, Göttingen, Germany; ^7^Institute for Cellular and Molecular Immunology, University Medical Center Göttingen, Göttingen, Germany

**Keywords:** adaptive NK cells, rhesus macaque (*Macaca mulatta*), NKG2A, NKG2C, Mamu-E, CMV

## Abstract

Previous research on adaptive NK cells in rhesus macaques suffered from the lack of specific antibodies to differentiate between inhibitory CD94/NKG2A and stimulatory CD94/NKG2C heterodimeric receptors. Recently we reported an expansion of NKG2C receptor-encoding genes in rhesus macaques, but their expression and functional role on primary NK cells remained unknown due to this deficit. Thus, we established monoclonal antibodies 4A8 and 7B1 which show identical specificities and bind to both NKG2C-1 and NKG2C-2 but neither react with NKG2C-3 nor NKG2A on transfected cells. Using a combination of 4A8 and Z199 antibodies in multicolor flow cytometry we detected broad expression (4-73%) of NKG2C-1 and/or NKG2C-2 (NKG2C-1/2) on primary NK cells in rhesus macaques from our breeding colony. Stratifying our data to CMV-positive and CMV-negative animals, we noticed a higher proportion (23-73%) of primary NK cells expressing NKG2C-1/2 in CMV+ as compared to CMV- macaques (4-5%). These NKG2C-1/2-positive NK cells in CMV+ macaques are characterized by lower expression of *IL12RB2*, *ZBTB16*, *SH2D1B*, but not *FCER1G*, as well as high expression of *IFNG*, indicating that antibody 4A8 detects CMV-associated adaptive NK cells. Single cell RNA seq data of 4A8-positive NK cells from a rhCMV-positive macaque demonstrated that a high proportion of these adaptive NK cells transcribe in addition to *NKG2C-1* and *NKG2C-2* also *NKG2C-3*, but interestingly *NKG2A* as well. Remarkably, in comparison to NKG2A, NKG2C-1 and in particular NKG2C-2 bind Mamu-E with higher avidity. Primary NK cells exposed to Mamu-E-expressing target cells displayed strong degranulation as well as IFN-gamma expression of 4A8+ adaptive NK cells from rhCMV+ animals. Thus, despite co-expression of inhibitory and stimulatory CD94/NKG2 receptors the higher number of different stimulatory NKG2C receptors and their higher binding avidity to Mamu-E outreach inhibitory signaling *via* NKG2A. These data demonstrate the evolutionary conservation of the CMV-driven development of NKG2C-positive adaptive NK cells with particular molecular signatures in primates and with changes in gene copy numbers and ligand-binding strength of NKG2C isotypes. Thus, rhesus macaques represent a suitable and valuable nonhuman primate animal model to study the CMV-NKG2C liaison *in vivo*.

## Introduction

An important and conserved family of NK cell receptors is represented by the C-type lectin-like receptor heterodimers CD94/NKG2A and CD94/NKG2C, which both specifically interact with the nonclassical MHC class I protein HLA-E in human. While the NKG2A protein is equipped with cytosolic immunoreceptor tyrosine-based inhibitory motifs and forms a typical inhibitory receptor, NKG2C lacks these motifs and instead associates with the immunoreceptor tyrosine-based activating motif-containing DAP12 adaptor protein that transmits stimulatory signals upon interaction of CD94/NKG2C with HLA-E. The inhibitory CD94/NKG2A is the first receptor that appears during differentiation of NK cells after hematopoietic stem cell transplantation (1) or in *in-vitro* differentiation assays (2). Besides NK also CD8+ T cells can express CD94/NKG2A and it has been shown that this receptor plays an important role in regulating the activity of these lymphocytes (3–5). Intimately linked with this function of NKG2A is stabilized cell-surface expression of HLA-E, which is effectively achieved through binding of hydrophobic peptides that are mostly derived from the leader peptides of other MHC class I proteins (6).

Subsets of NK cells can differentiate into cells displaying features of immunological memory such as antigen-specific activation, clonal expansion, or longevity, and are known as adaptive NK cells (7–12). The development of adaptive NK cells is driven by infections with viruses, in particular persisting viruses. Infections with the β-herpesvirus cytomegalovirus (CMV) in humans frequently elicit clonal expansions of NK cells expressing the stimulatory heterodimeric CD94/NKG2C receptor and an inhibitory KIR that is specific for the individual’s HLA-C class I protein (13–15). This expansion is driven by interaction between CD94/NKG2C and its ligand HLA-E, which can also present a peptide derived from the UL40 glycoprotein of HCMV (16, 17). Recognition of such UL40-peptide presenting HLA-E induces degranulation in NKG2C+ NK cells (17). HCMV infection not only impacts the NK cell repertoire by clonal expansion of NKG2C+ NK cells, but results also in altered function of these cells as evidenced by low expression of IL12RB2, IL18RAP, ZBTB16, FCER1G, SYK and SH2D1B (9, 18). Similar expansions of adaptive NK cells carrying a stimulatory receptor were identified in the mouse, where Ly49H+ NK cells expand upon infection with mouse CMV and specifically kill virally infected cells (10).

In contrast to the single human *NKG2C* gene, rhesus macaques are characterized by an expansion of *NKG2C* genes (19). While the NKG2C-1 and NKG2C-2 proteins are very similar (96% identical), the NKG2C-3 protein exhibits 91-92% identity to the two other NKG2C isotypes. Yet, the expression, the function, and the role in CMV infection of these three NKG2C isotypes was hitherto unknown. Here we report the establishment of monoclonal antibodies that specifically react with both rhesus macaque NKG2C-1 and NKG2C-2 and are suitable to detect adaptive NK cells in these nonhuman primates. Similar to human, rhesus macaque adaptive NK cells are characterized by expansion of NKG2C1/2-expressing NK cells due to rhCMV infection. Further, these adaptive NK cells show reduced expression of cytokine receptors and signal transduction factors, indicating that impact of CMV infection on NK cells is largely conserved in primates. We also present functional data of these receptors in primary rhesus macaque NK cells upon binding to their ligand Mamu-E as well as binding studies using a recombinant single-chain trimer Mamu-E protein.

## Materials and methods

### Rhesus macaque samples

Rhesus macaques (*Macaca mulatta*) kept at the German Primate Center under human care are undergoing a yearly routine veterinary health control that includes sampling of peripheral blood. Small aliquots of these samples were obtained from the Animal Husbandry Unit for diagnostic phenotyping. The collected peripheral blood samples were used to isolate PBMCs (peripheral blood mononuclear cells) following the procedures as described earlier by us (20). In addition, a single spleen sample from autopsy of a euthanized animal (rhCMV+) was obtained from the Pathology Unit. The pieces of the spleen were minced and homogenized in dissociation buffer (1X PBS + 1 mM EDTA) before gently passing through a cell strainer (pore size 70 µm). The mesh was washed using washing buffers (1X PBS + 2% FBS) and the suspension was collected in buffers to perform centrifugation (300 x g for 10 min at RT), and an additional washing step was done before collecting the pellet. Isolated PBMCs or single-cell suspensions from spleen were stored at -140 °C or incubated overnight in mediumPBMC1 (RPMI-1640 + GlutaMax, 10% inactivated FBS, 0.1% Gentamicin) or mediumPBMC2 (RPMI-1640 + GlutaMax, 10% inactivated FBS, 0.1% Gentamicin, 500 U/ml IL-2 and 10 ng/ml IL-15) prior to the experiment at the next day.

### Expression constructs used in this study

The open reading frames (ORF) of rhesus macaque CD94, NKG2A, NKG2C-1, NKG2C-2, NKG2C-3 were cloned in an expression vector containing the EF1a promoter. The ORFs of the NKG2C receptors were extended by a P2A sequence and the myc-tagged DAP12. T2A sequences were used to express proteins conferring puromycin resistance (for CD94) or blasticidin resistance (for NKG2A, NKG2C-1, NKG2C-2, NKG2C-3). For expression of rtTA3 we used Addgene plasmid #26429. The single-chain Mamu-E-B2M-peptide (Mamu-E trimer) open reading frame was synthesized by a commercial provider and cloned in a vector to extend the trimer sequence by a T2A sequence and blasticidin resistance and under the control of a tetracycline-inducible promoter. The region encoding the extracellular part of the Mamu-E trimer was amplified and cloned in the pFUSE-hIgG1-Fc2 vector (*In vivo*gen). An overview of the constructs is shown in **Figure S1**.

### Establishment of mouse anti-rhesus macaque NKG2C-1/C-2 monoclonal antibodies

We established a recombinant protein consisting of the extracellular part of rhesus macaque NKG2C-2 protein fused to the Fc domain human IgG1 using the pFUSE-hIgG1-Fc2 vector (*Invivo*gen). Production and purification of the Fc fusion protein, immunization of mice and establishment and selection of hybridoma cells were exactly as described in Rosner et al. (21) and Hermes et al. (22). Permission for the immunization of mice was obtained from the responsible authority, the Lower Saxony State Office for Consumer Protection and Food Safety, permit number 33.42502-05-A-029/09. Supernatants of hybridoma clones were tested in ELISA against the NKG2C-2 Fc protein and against human IgG to identify clones reacting with only NKG2C-2. Hybridoma cell clones 4A8 and 7B1 showed strong reaction only against the NKG2C-2-Fc protein and were further propagated in medium_H_ (RPMI-1640 + GlutaMAX, 10% inactivated FBS, 0.1% Gentamicin). For isolation of monoclonal antibodies, the medium of growing hybridoma cells was replaced with serum-free medium ProCHO 4 Protein-Free CHO Medium (Biozym Scientific) and the cells were cultured at 37°C with 5% CO_2_ for 2-3 days. The antibody-containing supernatant was collected, centrifuged for 5 min at 1300 x g, and filtrated with a 0.45 µm Minisart Syringe Filter (Sartorius). Isolation of the monoclonal antibodies was performed by affinity chromatography using HiTrap Protein G HP columns according to the recommendations of the supplier (GE Health Care Life Sciences). The volume of the solution containing the eluted monoclonal antibody was reduced using Amicon Ultra Centrifugal filters (Merck Millipore). The final concentration of the purified monoclonal antibodies was measured with a Qubit 4 Fluorometer (Thermo Fisher Scientific) using Qubit Protein Assay Kits (Thermo Fisher Scientific).

### Transfection of cells

HEK-293 cells were transfected with plasmid DNA using Lipofectamine 2000 according to the instructions of the supplier (Thermo Fisher Scientific). Plasmid DNA was also nucleofected into YT, NK-92 or 721.221 cells using the Nucleofector II (Lonza) electroporation-based transfection system according to the manufacturer’s instructions. Transfected cells were cultured at 37°C with 5% CO_2_ for at least 48 hr before further experiments were performed. Stably transfected cells were obtained by selection with the plasmid DNA specific antibiotic for at least 14 days. HEK-293, YT, NK-92 or 721.221 cells were cultured in medium_HEK293_ (DMEM + high Glucose + Stable Glutamine + 25 mM HEPES, 10% inactivated FBS and 0.1% Gentamicin), medium_YT_ (RPMI-1640 + GlutaMAX, 20% inactivated FBS, 55 μm beta-mercaptoethanol and 0.1% Gentamicin), medium_NK92_ (Alpha MEM + Ribonucleosides + Deoxyribonucleosides, 12.5% Horse serum, 12.5% inactivated FBS, 2 mM L-Glutamine, 0.1% Gentamicin and 200 U/ml IL-2) or medium_721_ (RPMI-1640 + L-Glutamin + 25 mM HEPES, 10% inactivated FBS and 0.1% Gentamicin), respectively.

### Detection of rhCMV status

Serum samples from rhesus macaque individuals were subjected to detection of rhCMV-specific immunoglobulin G (IgG). Detection was performed according to the instructions of the Simian Panel E Kit (Intutive Biosciences USA). All rhesus macaque blood donors used in this study were tested as described previously (23).

### Multicolor flow cytometry analysis of transfected cells and of PBMCs

Overnight grown HEK-293, YT or NK-92 cells (1 × 10^6^) expressing rhCD94 and either NKG2A, NKG2C-1, NKG2C-2, or NKG2C-3 with rhDAP12 (**Figure S1**) were harvested using pre-warmed (at 37°C) PBS and resuspended in 100 μl staining buffer_1_ (PBS, 0.5% BSA and 0.05% sodium azide). Resuspended cells were treated with 2 μl APC-conjugated mouse anti-human NKG2A (clone Z199) antibody (Beckman Coulter Life Sciences), 1 μl Alexa fluor 700-conjugated mouse anti-human NKG2C (either clone 134591 or 134522; R&D System) antibody or 1 μg purified mouse anti-rhesus macaque NKG2C-1/2 (either clone 4A8 or 7B1) primary antibody for 40 min at RT. After binding of 4A8 (or 7B1), cells were washed with PBS and 1.5 μl goat anti-mouse IgG conjugated with APC (BioLegend) secondary antibody was added in 100 μl staining buffer_1_ for 30 min at RT. Cells were then washed twice with PBS and resuspended with 100 µl live/dead cells staining buffer_Z_ (1:500, Zombie Aqua, BioLegend) for 10 min. After washing with PBS, cells were resuspended in PBS and transferred to 5 ml FACS tube (Sarstedt) for flow cytometry.

About 1 – 5 × 10^6^ rhPBMCs (overnight incubated in medium_PBMC1_) were prepared in 100 μl staining buffer_1_ with clone 4A8 antibody followed by PerCP-Cy5.5 or APC or PE-conjugated goat anti-mouse IgG (BioLegend) secondary antibody. Subsequently, FITC-conjugated mouse anti-human lineage markers antibodies CD3 (1:100; BD Biosciences), CD20 and CD14 (1:100; BioLegend), and 5 µl APC conjugated Z199 antibody in 100 µl of live/dead cells staining buffer_Z_ were applied for 30 min at RT. Anti-human NKG2C antibody clones 134591 and 134522 were also used for staining rhesus macaque PBMCs to check cross-reactivity of these antibodies. For staining rhesus macaque killer cell immunoglobulin-like receptors (KIRs), rhesus macaque pan-KIR3D antibody (clone 1C7) was used in rhPBMCs as described earlier by us (22, 24).

Tetracycline-inducible single-chain Mamu-E trimer (Mamu-E+B2M+peptide)-expressing 721.221 cells were stained with mouse anti-human HLA-E (clone 4D12) antibody (MBL Lifescience) or mouse anti-human HLA class I antibody (clone W6/32) and APC-conjugated goat anti-mouse IgG secondary antibody, respectively.

Transfected HEK-293 or YT cells expressing either NKG2C-2 (short stalk) or NKG2C-2L (long stalk) were also stained with clone 4A8 and a secondary antibody (APC conjugated goat anti-mouse IgG).

Flow cytometry experiments were performed using BD LSR II (BD Biosciences) or Spectral cell analyzer ID7000 (Sony). FlowJo version 10.8.0 in Mac OS X operating system was used to analyze the data. The Mean Fluorescence Intensity (MFI) was also calculated using FlowJo excluding the autofluorescence of each experimental setup.

### Cell sorting

Staining for cell sorting was performed following the same protocol as described above. Only sorting buffer (PBS, 2% FCS and 2 mM EDTA) was applied instead of PBS. From stained rhPBMCs, the populations of CD3-CD14-CD20- (lineage negative, lin- NK) and lin-4A8+ cells were sorted using a SH800 cell sorter (Sony) and collected into medium_PBMC1_.

### Comparative qRT-PCR

Fractions of lin- NK cells from PBMCs of rhCMV+/- rhesus macaques were sorted for subsequent RNA extraction using the Universal RNA/miRNA Purification Kit according to the manufacturer’s (Roboklon) protocol. Isolated RNA was used to generate cDNA following the manufacturer’s protocol of Transcriptor High Fidelity cDNA Synthesis KIT (Roche Diagnostics). A comparative qPCR was performed using SsoFast Evagreen Supermix with Low Rox (Bio-Rad) applying 40 cycles with initial denaturation at 95°C for 10 min, denaturation at 95°C for 15 sec, annealing at 58°C for 15 sec, extension at 72°C for 15 sec, and after 95°C for 15 sec, 1 min at 60°C for reading fluorescence with dissociation at 95°C for 1 sec using the Quant Studio 3 System (Applied Biosystems). Data obtained from the comparative qPCR were analyzed in Quant Studio Design and Analysis Software Version 1.5.1, and 2^-ΔΔCt method (25) was used for measuring expression or fold change. Rhesus macaque *RPL13A* was used as the housekeeping gene. All the primers of target genes evaluated in this study are listed in **Table S1**.

### Confocal laser microscopy

Transfected HEK-293 cells expressing NKG2C-2 or NKG2C-2long stalk (NKG2C-2L) with rhCD94+rhDAP12 were incubated in 100 μl staining buffer_1_ with 1 μl APC-conjugated mouse anti-human NKG2A (clone Z199) antibody for 40 min at RT. Cells were washed two times with PBS and subsequently Fluoromount-G Mounting Medium with DAPI (Thermo Fisher Scientific) was applied to the cells on a glass slide (Carl Roth). Samples were incubated for 5 min at RT and covered with a 24 x 24 mm cover slip (Carl Roth). Slides containing immunolabelled cells were analyzed under a confocal laser microscope LSM 800 (Carl Zeiss) equipped with ZEN 2.3 software (Carl Zeiss) and images were captured with Plan-Apochromat 63X/1.40 oil objective. The MFI value of Z199 was calculated in a fixed circular area of individual cells and images were processed using ZEN 2.3 software and ImageJ version 1.53e for Windows.

### Degranulation assay and induction of IFN-gamma

Tetracycline inducible single-chain Mamu-E trimer expressing 721.221 cells were stimulated with (1 μg/ml) or without doxycycline (dox) for 24 hr in medium_721_ and isolated rhPBMCs in medium_PBMC2_ with overnight incubation at 37°C with 5% CO_2_. A 1: 2 cells interaction ratio (721.221 cells: rhPBMCs) was then placed into the wells of a 48-well plate in 400 µl medium_PBMC1_ and cells were incubated at 37°C with 5% CO_2_. Ten µl of PE-conjugated mouse anti-human CD107a antibody (BD Biosciences) was also added to each treated well. After 1 hr incubation, 1X Monensin golgistop and 1X Brefeldin A (1000X stock, BioLegend) were added and incubated for 5 hr. Cells of each treated well were washed with PBS and centrifuged (500 x g, 5 min) and subsequently stained with mouse anti-human CD3, CD14, CD20 or NKG2A (clone Z199) antibodies and mouse anti-rhesus NKG2C-1/2 (clone 4A8) antibody following the same protocol as described above. CD107a was determined in the lin- NK or within lin- NK in the 4A8+Z199+ or 4A8-Z199+ NK subpopulations of stained rhPBMCs obtained from rhCMV+ and rhCMV- donors. Following the same protocol, induction of IFN-gamma was also measured in rhPBMCs upon 6 hr stimulation with dox-inducible Mamu-E trimer expressing 721.221 cells. After the staining, cells were fixed with Fixation buffer (BioLegend) and permeabilized using 1X Intracellular Staining Permeabilization Wash Buffer (BioLegend). Permeabilized cells were incubated for 10 min in blocking buffer (0.5% BSA and 2% normal fetal bovine serum in PBS) prior to addition of 3 μl PE-conjugated anti-human IFN-gamma antibody (BioLegend) and 30 min incubation at RT. Cells were washed twice with PBS, resuspended in PBS and transferred into tubes for flow cytometry. Induction of CD107a was also measured in YT or NK-92 cells during 6 hr stimulation with or without dox-induced Mamu-E trimer-expressing 721.221 cells. Flow cytometry experiments were performed in the Spectral cell analyzer ID7000.

### Interaction assays between NKG2 receptors and Mamu-E

The Mamu-E trimer-Fc construct (see **Figure S1**) was stably transfected into HEK-293 cells. Three days prior to harvest, the cells were incubated in ProCHO 4 serum-free medium (Lonza). The supernatant was then subjected to affinity chromatography using HiTrap Protein G HP columns to isolate and purify the Mamu-E trimer Fc protein. The protein concentration was measured in a Qubit 4 Fluorometer using Qubit Protein Assay Kits. An equal number of stably transfected HEK-293 cells expressing NKG2A, NKG2C-1, NKG2C-2, or NKG2C-3 were resuspended with 1 µg purified Mamu-E trimer Fc fusion protein in 100 µl PBS and incubated for 1 hr at RT. Cells were then washed two times in PBS and 3 µl of APC-conjugated mouse anti-human IgG-Fc secondary antibody (BioLegend) was applied for 30 min at RT within 100 µl PBS. After the incubation, cells were washed again three times and transferred to a tube containing PBS for flow cytometry. In parallel, these cells were also stained with clone Z199 antibody following the same protocol described above. The MFI of Mamu-E trimer Fc-bound cells and the MFI of clone Z199 antibody-bound cells were calculated (excluding autofluorescence). In order to take into account differences in the expression of NKG2 receptors in the different transfected cells, we normalized the binding of the Mamu-E-Fc protein. For this, the MFI of Mamu-E trimer-Fc was divided by the MFI of antibody Z199. This ratio for NKG2A, NKG2C-1, NKG2C-2, and NKG2C-3 were plotted in a graph. An increase in the ratio means a higher avidity of the soluble Mamu-E-trimer-Fc protein for this particular receptor.

### Co-immunoprecipitation and western blot

An equal number of stably transfected HEK-293 cells expressing CD94 and either NKG2C-2 or NKG2C-2L and untransfected HEK293 cells were cultured in medium_HEK293_ at 37 °C with 5% CO_2_. Cells (90 – 95% confluence) were washed with ice cold PBS and lysed in ice cold lysis buffer (10 mM Tris-HCl pH 7.5, 150 mM NaCl, 1% N-Dodecyl beta-D-maltoside (Thermo Fisher Scientific), 0.4 mM EDTA and 1 tablet protease-inhibitor-cocktail complete mini (Roche Diagnostic)) with 20 min gentle shaking at 4°C. The lysate was collected after the centrifugation (15 min, 16000 x g), and an equal amount of lysate was pre-cleared using Protein G sepharose beads (GE healthcare). 20 μg of pre-cleared lysate was incubated with 2.5 μg of antibody clone 4A8 overnight at 4°C with rotation, and then the protein mixture was added to the Protein G sepharose beads for 5 hours at 4°C with rotation. A centrifugation at 10000 x g at 4°C was performed and beads were washed with washing buffer (10 mM Tris-HCl pH 7.5, 150 mM NaCl, 1% N-Dodecyl beta-D-maltoside). Finally, the bound proteins were eluted from the beads using 5X SDS PAGE reducing protein loading buffer (Bosterbio) at 95°C for 5 min and centrifuged (5 min, 10000 x g at RT). The protein content in the supernatant was measured with a Qubit 4 fluorometer using the Qubit protein assay kit (Thermo Fisher Scientific).

Equal amounts of the two lysate samples and the two Co-IP samples were separated in 8 – 16% Mini protean-TGX stain free protein gels (Bio-Rad) and blotted on Trans-Blot Turbo mini PVDF membranes (Bio-Rad) using the Trans-Blot Turbo Transfer system (Bio-Rad). Everyblot Blocking Buffer (Bio-Rad) was applied to the blot for 10 min with gentle shaking. The myc-tagged DAP12 of the lysate or Co-IP sample was detected using rabbit anti-myc primary antibody (1:2000 dilution within blocking buffer; Abcam) and a secondary goat anti-rabbit immunoglobulin antibody conjugated with horse radish peroxidase (HRP) (1:2500 dilution within blocking buffer; Santa Cruz Biotechnology). Finally, Immobilon Forte Western HRP Substrat (MerckMillipore) solution was applied to the blot for the chemiluminescent detection. The image of the blot and the intensity of the band were analysed using software ImageJ2 version 2.9.0/1.53t for Mac Os.

### Single cell RNA sequencing and bioinformatic analysis

Lin-4A8+ cells were sorted from a rhCMV+ rhesus macaque for single cell RNA sequence analysis using the single cell RNA-seq platform iCELL8 (Takara) combined with the single cell dispenser and imaging system CellenONE X1 (Cellenion) essentially as recently described by one of our co-authors (26). High-throughput sequencing of the single cell libraries was performed using an Illumina HiSeq4000.

For preprocessing of single cell data, the *Macaca mulatta* reference genome Mmul 10 (Ensembl release 105) was manually reviewed and curated to include in-house annotations of *NKG2* and *KIR* genes and encompassed *KLRC1* (*NKG2A*), *rhKLRC2* (*NKG2C-1*), *rhKLRC3* (*NKG2C-2*), *rhKLRC4* (*NKG2C-3*), *rhKLRC5* (*NKG2F*), *KIR1D*, *KIR2DL04*, *KIR3DL01*, *KIR3DL01*, *KIR3DL07*, *KIR3DL08*, *KIR3DL11*, *KIR3DL20*, *KIR3DS02*, *KIR3DS05*, *KIR3DS06*, *KIR3DSW07* and *KIR3DSW09*. Manual curations were based on sequence alignments of publicly available coding sequences available from the IPD-NHKIR database and *NKG2A*, *NKG2C-1/2/3* and *NKG2F* coding sequences as previously published by our group (19). This custom version of the reference genome along with read data and the generated Seurat object in RDS format are available in Gene Expression Omnibus (GEO) under accession number GSE211613. Reads were aligned to the custom reference genome using STAR version 2.7.10a (27). The STAR option “–quantMode GeneCounts” was used to generate the count matrix. The quality of reads and STAR alignments was evaluated using FastQC v0.11.9 (available at www.bioinformatics.babraham.ac.uk/projects/fastqc) and summarized using MultiQC version 1.0.dev0 (28).

Analysis of single cell data was performed using Seurat version 4.1.0 (29) under Ubuntu 20.04 LTS and R version 4.1.2 (www.r-project.org). Our quality control and filtering of cells was guided according to recommendations in the Seurat manual and published reports (30). Cells with more than 3% mitochondrial reads, more than 50% ribosomal reads, less than 200 or more than 6000 features, with less than 50% mapped reads, or cells with one feature constituting more than 25% of the reads were excluded from the analysis. A quality assessment is shown in **Figure S2A**. After these filtering steps, 733 cells remained for analysis. Principal component analysis (PCA) and cell cycle scoring were performed using Seurat. Cell cycle stage had no impact on clustering or PCA (**Figure S2B**). TPMs were calculated using the ,,calculateTPM” function from the scuttle R package version 1.4.0 (31) and were put on the log scale using log_e_(1+x)). Gene ranges for TPMs were calculated as exon sums from the custom *Macaca mulatta* annotation file using the GenomicFeatures R package version 1.46.1 (32). TPMs were used as normalized values in all downstream analyses. The variable ,,sample wells” was regressed out using the linear model provided in Seurat’s ,,ScaleData” function. The first 25 principal components were used for clustering and UMAP visualizations.

UpSet plots (33) were created using the UpSetR R package version 1.4.0 (34). The cutoff for the distinction of “expressed” and “not expressed” was 0.5 log scaled TPM. The R packages Seurat, ggplot2 version 3.3.5, ggridges version 0.5.3 and patchwork version 1.1.1 (all available at cran.r-project.org) were used for preparing figures.

### Statistical analysis

Statistical analyses were performed with GraphPad Prism 9. Student’s t-test (parametric, unpaired/paired, two-tailed, 95% confidence level) considering p values >0.05 as not statistically significant was maintained to analyze differences between groups. For comparisons of Mamu-E-Fc protein binding to NKG2 receptors, we asked whether NKG2C-1, NKG2C-2, and NKG2C-3 bind differently from NKG2A and applied one-way ANOVA with Dunnett’s multiple comparisons test and used NKG2A as control.

## Results

### Expression of expanded rhesus macaque NKG2C genes and establishment of monoclonal antibodies

**Figure 1:**
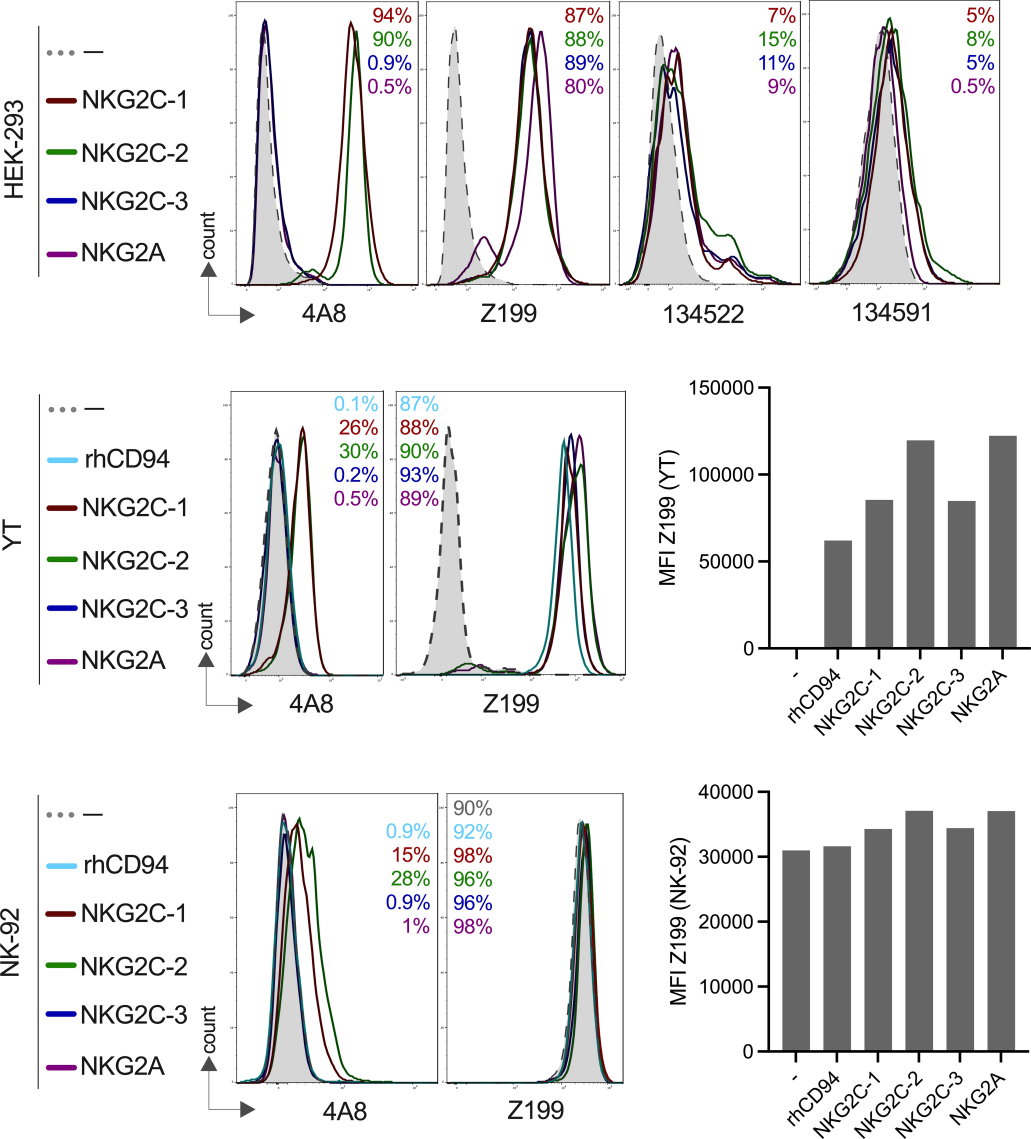
Expression of rhesus macaque CD94/NKG2 receptors and specific binding of monoclonal antibodies. HEK-293, YT or NK-92 cells were co-transfected to express either rhesus macaque NKG2A, NKG2C-1, NKG2C-2 or NKG2C-3 with rhCD94+rhDAP12+/-. Staining was performed using anti-rhesus macaque NKG2C-1/2 antibody 4A8, anti-human NKG2A antibody Z199, anti-human NKG2C antibody clones 134522 and 134591 for the indicated cell lines and transfections. Percentage of positive cells are indicated in the histogram plots and are in the same order and same color as shown on the left for the gene constructs. Bar graphs show the calculated MFI of Z199+ cells in transfected NK-92 or YT cells.

We recently reported the genomic organization of the rhesus macaque killer cell lectin-like receptor region where we identified three NKG2C-encoding genes, and single-copy *NKG2A* and *NKG2F* genes (19). In order to study their cell surface expression and specificity for antibodies, we expressed *NKG2A* and the three *NKG2C* genes together with rhesus macaque CD94 and in the case of NKG2C1-3 also with the rhesus macaque DAP12 adaptor protein in HEK-293 cells (an overview of the constructs is shown in **Figure S1**). As can be seen in **Figure 1**, all rhesus macaque NKG2 proteins were expressed on the cell surface of HEK-293 cells and react with the anti-human NKG2A antibody Z199. These data confirm and extend previous findings by Biassoni et al. (35) and LaBonte et al. (36) who found cross-reaction of antibody clone Z199 with rhesus macaque NKG2A and NKG2C (the authors used a single NKG2C-encoding construct). We also included in our analysis two monoclonal anti-human NKG2C antibodies (clones 134522 and 134591). A very low level or no reaction was found using these antibodies with rhesus macaque PBMCs (**Figure S3**). Using the single transfectants, antibody clone 134522 reacted on very low levels with all three CD94/NKG2C isotypes as well as with CD94/NKG2A and showed, therefore, the same binding pattern as antibody clone Z199, while antibody clone 134591 failed to react with NKG2A, and showed very low binding to all three NKG2C receptors (**Figure 1**) and failed to react with lin-NK cells of PBMCs (**Figure S3**).

As there is currently no antibody available to clearly distinguish NKG2A and the three NKG2C proteins in rhesus macaques, we decided to generate monoclonal antibodies. We produced a recombinant NKG2C-2 protein tagged with the Fc part of human IgG1 and used this for immunization of mice. Antibody-producing hybridoma cell clone 4A8 showed an interesting reaction pattern and specifically reacted with both NKG2C-1 and NKG2C-2, but neither with NKG2C-3 nor NKG2A on transfected HEK-293, YT and NK-92 cells (**Figure 1**). A further antibody clone, 7B1, showed exactly the same pattern as 4A8 (not shown). Thus, we established two mouse monoclonal antibodies that can specifically detect stimulatory C-type lectin-like receptors CD94/NKG2C-1 and CD94/NKG2C-2 in rhesus macaques. As both clones reveal identical specificity, we focused our studies on clone 4A8.

For the subsequent functional analyses of the rhesus macaque NKG2 receptors, we also transfected the corresponding constructs in the human NK cell lines NK-92 and YT. NK-92 cells display endogenous expression of human CD94/NKG2A, whereas YT cells express only endogenous human NKG2A, but not human CD94 (37), and, therefore, parental YT cells are negative with antibody Z199. Our data confirmed these endogenous expression patterns (**Figure 1**). Upon transfection of YT cells with rhesus macaque CD94, the endogenous human NKG2A paired with rhCD94 and this human-rhesus chimeric receptor can be detected on the cell surface with antibody Z199 (**Figure 1**). These endogenous expression of NK-92 and YT cells have to be taken into account when rhesus macaque expression constructs were tested. Therefore, an increase in the MFI of Z199 should reflect the successful cell surface expression of rhesus macaque NKG2A, NKG2C-1, NKG2C-2 or NKG2C-3 in NK-92 or in rhCD94+ YT cells. Indeed, the MFI significantly increased after co-transfection of each of the rhesus macaque NKG2 constructs along with rhCD94 and in case of the NKG2C isotypes also with rhDAP12 (**Figure 1**), indicating successful expression of these rhesus macaque CD94/NKG2 receptors in both NK-92 and YT cells.

In summary, we have demonstrated clear cell surface expression of NKG2A, NKG2C-1, NKG2C-2, and NKG2C-3 as heterodimers with CD94 on transfected cells. Our newly established antibodies 4A8 and 7B1 specifically react with CD94/NKG2C-1 and CD94/NKG2C-2, but neither with CD94/NKG2C-3 nor CD94/NKG2A.

### Expression of NKG2A, NKG2C-1, NKG2C-2, NKG2C-3 in primary NK cells of rhCMV+ and rhCMV- rhesus macaques

**Figure 2:**
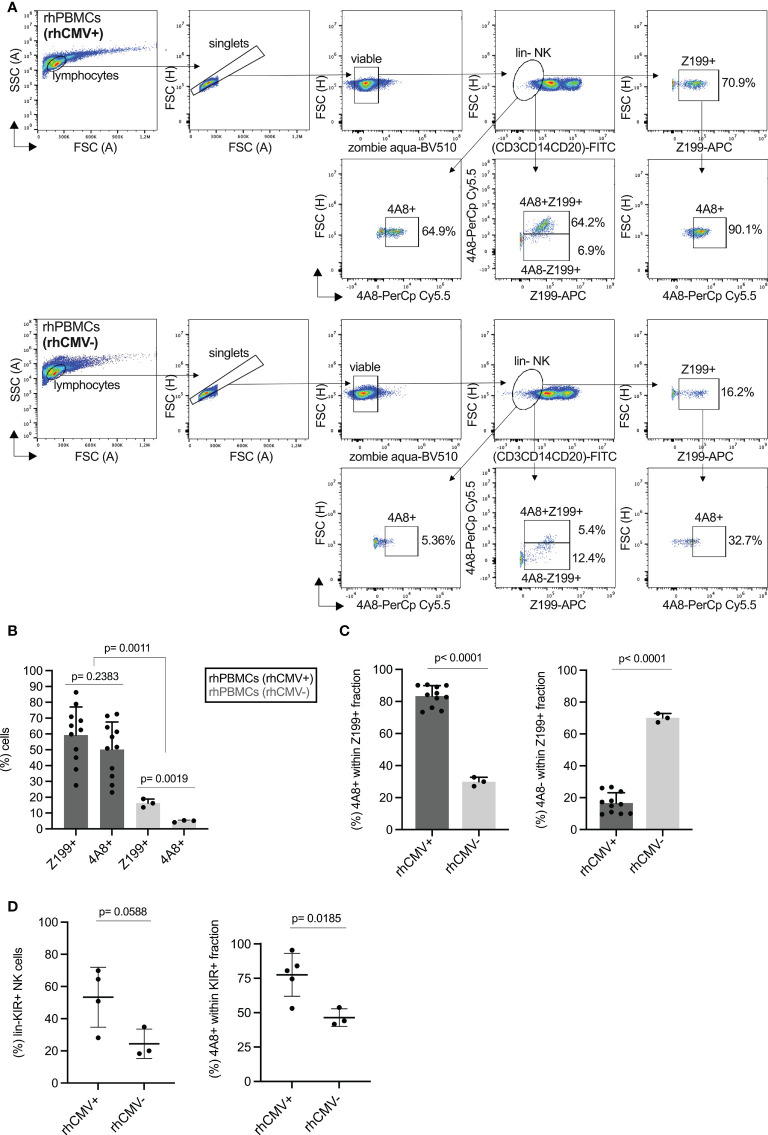
Expression of rhesus macaque NKG2C-1/2 in NK cells of CMV- and CMV+ rhesus macaques. **(A)** Gating strategy of PBMCs of CMV+ and CMV- rhesus macaques stained with Z199, 4A8 and lineage marker cocktail (CD3, CD14, CD20), and analysed in flow cytometry. **(B)** Percentage of Z199+ or NKG2C-1/2+ (4A8+) cell populations in CMV+ and CMV- rhesus macaques. One sample was not derived from peripheral blood but from spleen of a rhCMV+ animal. **(C)** Percentage of 4A8+ or 4A8- cell fractions were calculated within the lin-Z199+ NK cell population. **(D)** Percentage of anti-rhesus macaque KIR antibody 1C7 (22) positive cell populations were compared between CMV+ and CMV- rhesus macaques (left panel). We also calculated the percentages of 4A8+ cells within the KIR+ fraction of NK cells (right panel).

Next, we were interested in analyzing the expression of rhesus macaque CD94/NKG2 receptors in PBMCs derived from animals that are either positive (rhCMV+) or negative (rhCMV-) for the rhesus macaque cytomegalovirus. Among the rhesus macaques in our breeding colony (n=570) almost all are rhCMV+ and only a minimal proportion is rhCMV-. We used antibody 4A8 in combination with Z199 and lineage markers in multicolor flow cytometry of PBMCs derived from randomly chosen rhCMV+ and three rhCMV- rhesus macaques of our colony to analyze expression of NKG2C-1 and NKG2C-2. NK cells were defined as negative for lineage (CD3, CD20, CD14), and from the lineage negative (lin-) NK cells were gated to only 4A8+ or only Z199+, and then 4A8+ within the Z199+ population, or 4A8+Z199+/4A8-Z199+ cells, respectively (see gating strategy in **Figure 2A**). Among the rhCMV+ animals, we found 28% to 86% of the NK cells reacting with Z199 and 23% to 73% with 4A8 (**Figure 2B**). In contrast, 14% to 19% of the NK cells were positive with Z199 and only 4% to 5% were 4A8+ in rhCMV- animals (**Figure 2B**). As shown in **Figure 1A**, all NKG2 receptors stained positive with 4A8 are also Z199 positive. Therefore, we calculated the percentages of 4A8+ and 4A8- NK cells in the population of Z199+ NK cells. In rhCMV+ rhesus macaques the percentage of 4A8+ NK cells among all Z199+ NK cells is high (73% to 90%), whereas the percentage of 4A8- NK cells is low (10% to 27%; **Figure 2C**). Vice versa, the percentage of 4A8+ NK cells among all Z199+ NK cells is low (27% to 33%) and the percentage of 4A8- NK cells is high (67% to 73%) in rhCMV- animals (**Figure 2C**). These differences between CMV-positive and negative animals are statistically highly significant (p=0.0011, **Figure 2B**; p<0.0001, **Figure 2C**). Thus, in the rhCMV- animals there is a lower proportion of NKG2C-1/2 (Z199+4A8+) expressing NK cells and a higher proportion of Z199+4A8- NK cells. We conclude from these data that this Z199+4A8- NK cell subpopulation lacks expression of NKG2C-1/2 and consists of CD94/NKG2A and/or CD94/NKG2C-3 expressing NK cells.

We also analyzed the KIR protein expression using our pan-KIR3D antibody 1C7 (22). A higher percentage of KIR3D+ NK cells was noticed in lin- NK cells of CMV+ as compared to CMV- rhesus macaques, but this difference slightly missed statistical significance (**Figure 2D**). However, when we gated on the 4A8+ NK cells (**Figure S4**), the majority of 4A8+ NK cells of CMV+ macaques express also a KIR3D receptor in contrast to 4A8+ NK cells of CMV- animals where fewer cells are KIR3D+ (p=0.0185; **Figure 2D**). This indicates that CMV infection not only results in more NK cells expressing the stimulatory CD94/NKG2C-1/2 receptors, but this subpopulation is also characterized by expression of KIR. Due to the enormous polymorphism of KIR genes in rhesus macaques and lack of specific antibodies, we were not able to unambiguously distinguish these KIR receptors and to examine if a particular KIR protein is prominent in the 4A8+ NK cells of an individual.

**Figure 3:**
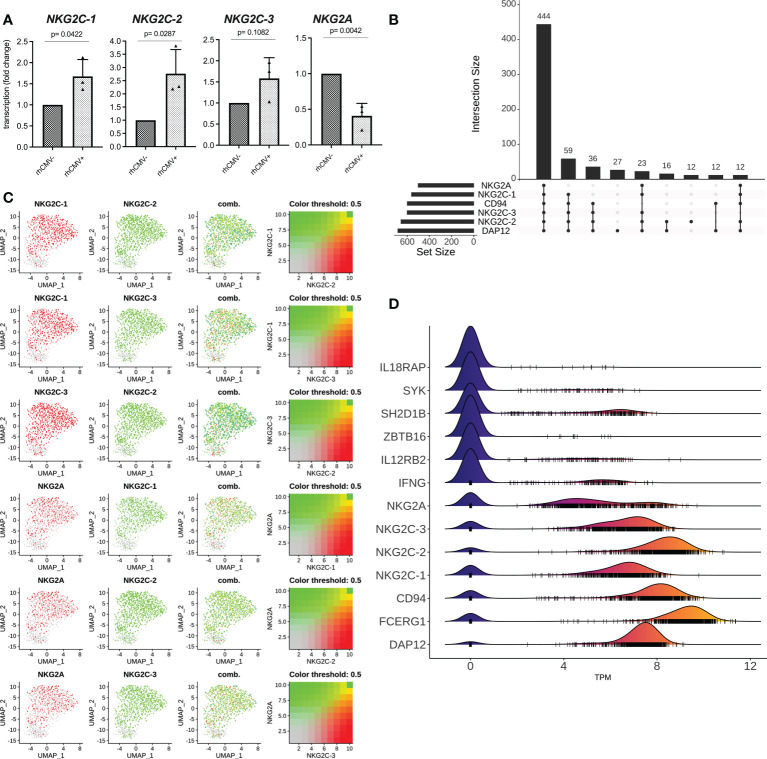
qRT-PCR and single cell RNA seq analysis. **(A)** Lin- NK cells were sorted from isolated rhesus macaque PBMCs and qRT-PCR was performed for genes *NKG2C-1*, *NKG2C-2*, *NKG2C-3*, and *NKG2A*. **(B-D)** Bioinformatic analysis of single cell RNA seq data from sorted lin-4A8+ NK cells isolated from a rhCMV+ rhesus macaque. **(B)** Upset plot showing the different combinations of receptor genes identified in single cells. The number of cells showing transcription of the individual combinations are indicated **(C)** UMAP plot showing the co-transcription of *NKG2A*, *NKG2C-1*, *NKG2C-2*, and *NKG2C-3*. **(D)** Ridgeline plot demonstrating the normalized transcript counts (TPM) of indicated genes. Small vertical lines indicate the TPM values of individual cells while ridges indicate the number of individual cells with similar transcript counts.

We also analyzed the transcription of the *NKG2* genes and established gene-specific primer pairs (**Table S1**) that were verified using the transfected HEK-293 cell panel (**Figure S5**). As can be seen in **Figure 3A**, the lin- NK cells of rhCMV+ animals displayed more *NKG2C-1* and *NKG2C-2* transcripts, whereas more *NKG2A* transcripts were detected in rhCMV- animals (**Figure 3A**). An increase was also noticed for *NKG2C-3* in rhCMV+ macaques, but lacked statistical significance (**Figure 3A**). In order to analyze the transcription of these receptor genes on single-cell level, we designed a small-scale experiment and performed single-cell RNA seq. Data obtained from sorted lin-4A8+ NK cells of a rhCMV+ rhesus macaque clearly demonstrate that the vast majority of lin-4A8+ NK cells simultaneously transcribe all four *NKG2* genes (**Figures 3B, C**). Analysis of the normalized transcript read counts (transcripts per million, TPM) of the various receptor genes revealed that *NKG2C-2* and *CD94* had the highest counts, followed by *NKG2C-1* and *NKG2C-3*, while *NKG2A* showed a dichotomous distribution with most of the single cells having fewer *NKG2A* transcripts and some having similar *NKG2A* read counts as *NKG2C-1* and *NKG2C-3* (**Figure 3D**).

These data collectively demonstrate that the majority of Z199+ NK cells in rhCMV+ animals are consisting of a population of NK cells expressing the stimulatory CD94/NKG2C-1 and/or CD94/NKG2C-2 receptors and that this high proportion is a consequence of rhCMV infection. Interestingly, the majority of 4A8+ NK cells transcribe all four NKG2 genes in addition to CD94 and should, therefore, be able to express the four different combinations of CD94/NKG2 receptors at the cell surface.

### Antibody 4A8 detects CMV-associated adaptive NK cells in rhesus macaques

**Figure 4:**
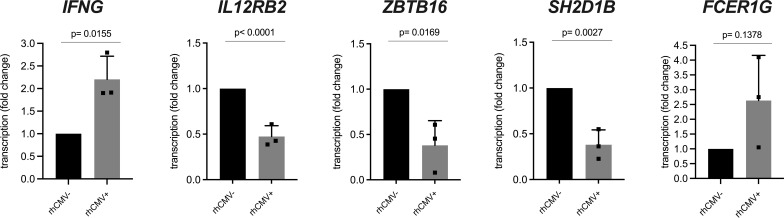
qRT-PCR analysis of lin- NK cells from rhCMV+ and rhCMV- rhesus macaques. Lin- NK cells were sorted using lineage markers (CD3, CD14, CD20) from isolated rhesus macaque PBMCs and qRT-PCR was performed for genes *IFNG*, *IL12RB2*, *SH2D1B*, *ZBTB16*, and *FCER1G* in comparison to housekeeping gene *RPL13A*.

In many HCMV-infected individuals, an increased frequency of NKG2C-expressing NK cells can be observed that is driven by this persisting viral infection (12, 13). This particular human NK cell subset is further characterized by longevity and functional changes such as decreased sensitivity to inflammatory cytokines IL-12 and IL-18, decreased expression of transcription factor ZBTB16 (PLZF) as well as signaling proteins SH2D1B (EAT-2) and FCER1G (FcR-gamma), increased expression of IFN-gamma and increased capability of performing ADCC (9, 18, 38, 39). This NK cell subset is referred to as CMV-associated adaptive NK cells. As we noticed an increase of NKG2C-1/2-expressing NK cells as a result of rhCMV infection, we asked whether NKG2C-1/2-positive NK cells show similar characteristics as human CMV-associated adaptive NK cells. We designed specific primers for genes *IFNG*, *IL12RB2*, *ZBTB16*, *FCER1G*, *SH2D1B*, and *FCER1G* (**Table S1**) and performed qRT-PCR using cDNA from sorted lin-NK cells of rhCMV+ and rhCMV- rhesus macaques. Indeed, the NK cells of rhCMV+ rhesus macaques showed less transcription of *IL12RB2* (p=0.0015), *ZBTB16* (p=0.0169) and *SH2D1B* (p=0.0027) and increased transcription of *IFNG* (p=0.0155) in comparison to NK cells of rhCMV- animals. For *FCER1G* we noticed an increase in rhCMV+ macaques which missed statistical significance (**Figure 4**). Contrasting human NK cells is the usage of the *FCER1G*-encoded FcR-gamma protein as the adaptor molecule for activating KIR in rhesus macaques (20). Therefore, downregulation of this adaptor may not be expected as this would thwart aKIR+ NK cells in rhesus macaques.

Thus, CMV-associated adaptive NK cells have very similar characteristics in humans and macaques and monoclonal antibody 4A8 in combination with Z199 is able to specifically detect this particular NK cell subset in rhesus macaques.

#### Interaction of rhesus macaque NKG2 receptors with Mamu-E

**Figure 5:**
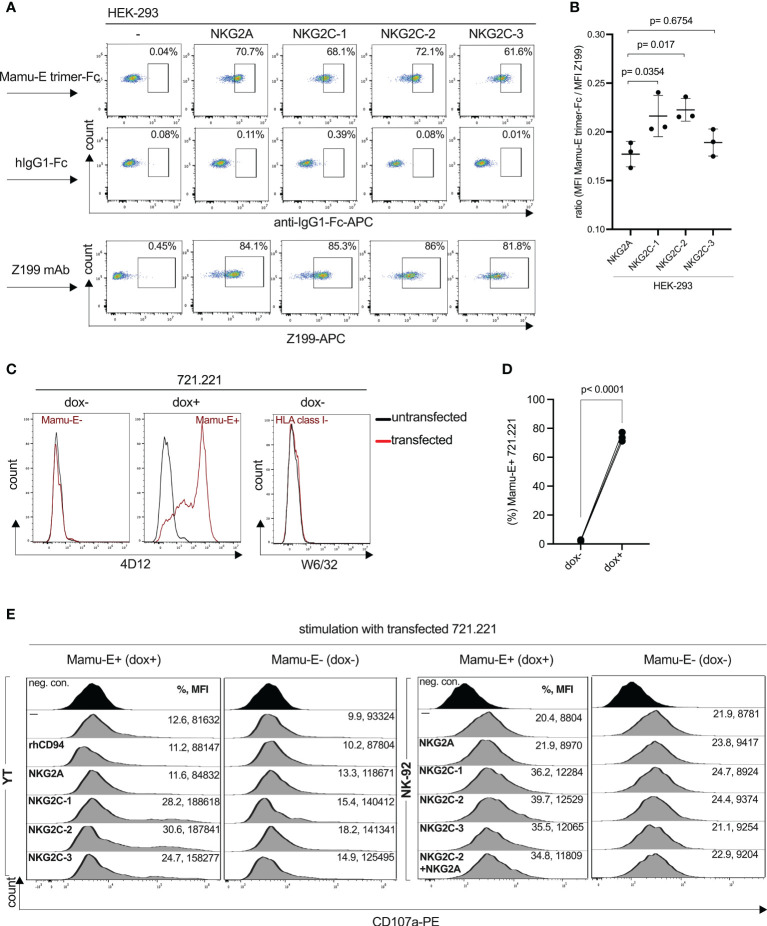
Interaction of rhesus macaque CD94/NKG2 receptors with Mamu-E. **(A)** Flow cytometry analysis of soluble single-chain Mamu-E trimer-Fc protein, control hIgG1-Fc protein or clone Z199 antibody stained HEK-293 cells transfected with the indicated gene constructs. Untransfected HEK-293 cells served as negative control (-). Percentages of positive cells are indicated. **(B)** Calculated ratio of the MFI of Mamu-E trimer Fc-bound cells divided by the MFI of Z199-bound cells of NKG2A, NKG2C-1, NKG2C-2 or NKG2C-3 is shown. Individual values and standard deviation are indicated. Statistical significance of differences of the mean were calculated using one-way ANOVA and Dunnett’s multiple comparisons test and used NKG2A as control. **(C)** Flow cytometry analysis of MHC-I-negative 721.221 cells, either untransfected or transfected with rtTA3 transactivator and doxycycline-inducible single-chain Mamu-E trimer (Mamu-E+B2M+VMAPRTLLL peptide) and stained with anti-human HLA-E antibody 4D12 (left and middle histogram) and left untreated (dox-) or treated with doxycycline (dox+) to induce the Mamu-E trimer, or stained with anti-human HLA class I antibody W6/32 (right histogram) and not treated with dox. **(D)** Percentage of 721.221 cells expressing the Mamu-E trimer upon treatment with doxycycline (dox+) or left untreated (dox-). **(E)** Degranulation of parental (–) YT and NK-92 cell lines or transfected with individual gene constructs (see **Figure 1**) and incubated with 721.221 cells either left untreated (Mamu-E-) or treated with dox to induce Mamu-E trimer expression (Mamu-E+). Percentage of CD107a+ cells and corresponding MFI are indicated.

Binding of the rhesus macaque CD94/NKG2 receptors to Mamu-E has been predicted, but to the best of our knowledge has not been formally shown and reported so far. For this, we produced a single-chain rhesus macaque Mamu-E (Mamu-E+beta-2-microglobulin+ rhesus MHC class I leader peptide VMAPRTLLL as a single chain linked by (GGGGS)_3_ and (GGGGS)_4_ sequences) protein fused to human IgG1-Fc. As negative control we produced only the Fc portion. The soluble proteins were incubated with HEK-293 cells expressing the individual NKG2C isotypes or NKG2A and analyzed by flow cytometry. As shown in **Figure 5A**, all four CD94/NKG2 receptors bind the soluble Mamu-E-Fc trimer protein. In order to compare the avidities of the NKG2 receptors for Mamu-E, the calculated MFI of Mamu-E-Fc binding was divided by the respective MFI of Z199 to take into account potential differences of receptor expression. According to the calculated mean ratio, the three stimulatory NKG2C isotypes showed stronger binding compared to the inhibitory NKG2A (**Figure 5B**). Specifically, the mean ratio was highest for NKG2C-2 (mean value 0.223), followed by NKG2C-1 (0.216) and NKG2C-3 (0.189), whereas NKG2A exhibits the lowest ratio (0.177). Notably, the statistical analysis revealed Mamu-E binding of NKG2C-2 and NKG2C-1 was different from NKG2A (p=0.0170, p=0.0354), whereas binding of NKG2C-3 was not (p=0.6754) (**Figure 5B**). After confirming the binding of Mamu-E trimer Fc proteins, we also used a construct for doxycycline (dox)-inducible expression of the Mamu-E trimer. This construct was stably transfected into 721.221 cells that were pre-transduced to express the rtTA3 transactivator protein. Addition of 1 µg/ml dox for 24 hr resulted in Mamu-E expression on 70% to 80% of 721.221 cells (**Figures 5C, D**). Without the addition of dox, the cells neither expressed Mamu-E nor other class I proteins (**Figure 5C**). Using this inducible Mamu-E expression system, we assessed the variability of ligand recognition of YT and NK-92 cells that were both transfected with the respective constructs. As functional readout of this recognition, we used degranulation assays *via* cell surface expression of CD107a (40). When one compares the percentages of CD107a-positive cells as well as the MFI of CD107a for each receptor interacting with Mamu-E+ and Mamu-E- 721.221 target cells, it became evident that dox-induced expression of Mamu-E resulted in clear increase of degranulation for the three NKG2C isotypes. In contrast, expression of NKG2A resulted in decreased degranulation (**Figure 5E**). The degranulation levels upon interaction of the three NKG2C isotypes with their ligand Mamu-E did not differ drastically, with NKG2C-2 showing the highest and NKG2C-3 the lowest values in both cell lines used.

We also measured degranulation upon transient co-expression of NKG2C-2 and NKG2A in NK-92 cells. Even in the presence of NKG2A we noticed a considerable increase in percentage of CD107a+ cells and CD107a MFI (**Figure 5E**). These data also suggests that CD94/NKG2C-2 binding of the Mamu-E ligand is stronger and that expression of CD94/NKG2A does not significantly inhibit degranulation in this experimental setting. Thus, NKG2C-2 and NKG2C-1 bind their ligand Mamu-E with higher avidity as compared to NKG2A. Interestingly, the opposite is true for human, where NKG2A binds HLA-E with higher avidity as compared to NKG2C (41).

#### Functional analysis of rhesus macaque adaptive NK cells

**Figure 6:**
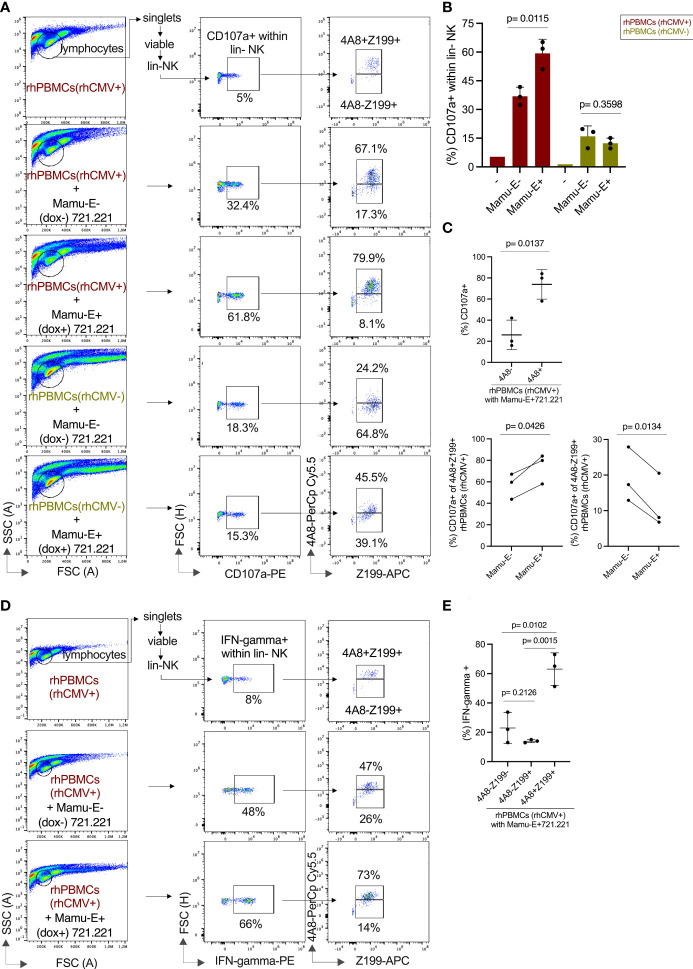
Functional analysis of NK cells of rhCMV- and rhCMV+ rhesus macaques upon interaction with Mamu-E. The flow cytometry analysis used antibodies against lineage markers, a combination of Z199 and 4A8 antibodies, anti-CD107a, and anti-IFN-gamma antibodies. **(A)** The gating strategy is indicated in brief and is shown in detail in **Figure S6B**. 721.221 cells left untreated (Mamu-E-) or upon induction of single-chain Mamu-E trimer by dox (Mamu-E+; see **Figure 5C**) were added at 1:2 ratio (721.221:PBMCs) for stimulation of PBMCs. Percentage of CD107a+ cells is indicated for the lin- NK cells (middle panels). Of these lin-CD107a+ NK cells we determined the fraction of NKG2C-1/2+ (4A8+Z199+) and NKG2C-1/2- (4A8-Z199+) NK cells (right panels). **(B)** Percentage of CD107a+ lin-NK cells are shown for untreated cells (-), for cells stimulated with 721.221 cells without dox induction (Mamu-E-), and for 721.221 after dox treatment to induce expression of single-chain Mamu-E (Mamu-E+). **(C)** The percentages of CD107a+ lin-4A8- (NKG2A+ and/or NKG2C-3+) NK cells and lin-4A8+ (NKG2C-1/2+) NK cells of rhCMV+ rhesus macaques upon interaction with Mamu-E+ 721.221 target cells are shown in the upper panel. The percentages of CD107a+ in the 4A8+Z199+ (NKG2C-1/2+) subpopulation or the 4A8-Z199+ (NKG2A+ and/or NKG2C-3+) subpopulation of lin-CD107a+ NK cells of rhCMV+ PBMCs upon interaction with Mamu-E- or Mamu-E+ target cells are shown in the lower panels. **(D)** Determination of IFN-gamma expression in lin-NK cells. The gating strategy is indicated in the left panels for untreated lin-NK cells (top panel), for lin-NK cells incubated with 721.221 cells without dox (Mamu-E-) (middle panel) or for lin-NK cells incubated with 721.21 cells treated with dox to induce single-chain Mamu-E trimer (bottom panel). The right panels show the percentages of 4A8+Z199+ and 4A8-Z199+ NK cells that express IFN-gamma. **(E)** The fraction of IFN-gamma+ cells of rhCMV+ rhesus macaques were calculated in the different lin- NK cell populations using antibodies 4A8 and Z199 upon interaction with 721.221 cells expressing dox-induced single-chain Mamu-E (Mamu-E+).

After showing the degranulation capacities of the CD94/NKG2 receptors in transfected cell lines, we analyzed PBMCs of rhCMV+/- donors to evaluate this function of 4A8+ adaptive NK cells. For this, we co-incubated PBMCs with Mamu-E-transfected 721.221 cells either induced by doxycycline (Mamu-E+) or left untreated (Mamu-E-), and performed CD107a assays and measured interferon-gamma. For these assays we stimulated the PBMCs overnight with IL-2 and IL-15, yet this cytokine treatment did not change the frequency of NK cells expressing NKG2C-1/2 (**Figure S6A**). The complete gating strategy of this multicolor flow cytometry using lineage markers, clone Z199, clone 4A8 and anti-CD107a antibodies is shown in **Figure S6B**. The baseline level of CD107a in the lin- NK cells of a rhCMV+ donor without stimulation by 721.221 cells was 5% (**Figure 6A** top). Upon interaction with Mamu-E- 721.221 cells, 32.4% of the lin- NK cells exhibited CD107a expression, indicating that the human 721.221 cells induce degranulation in rhesus macaque NK cells. Of these 32.4%, 67.1% expressed NKG2C-1/2 (4A8+Z199+) and 17.3% were NKG2C-1/2- (4A8-Z199+) NK cells. Interaction with Mamu-E+ 721.221 cells resulted in strong degranulation (61.8%) of the lin- NK cells, with 79.9% of these cells expressing NKG2C-1/2 (4A8+Z199+) NK cells and 8.1% being NKG2C-1/2- (4A8-Z199+) NK cells (**Figure 6A**). Overall, lin- NK cells of rhCMV+ rhesus macaques show an increased degranulation against Mamu-E+ 721.221 cells (p=0.0115; **Figure 6B**). After comparing the degranulation in the NKG2C-1/2+ (4A8+Z199+) and NKG2C-1/2- (4A8-Z199+) subpopulations (**Figure 6C**), it is clear that the 4A8+ adaptive NK cells of rhCMV+ animals were mainly responsible for the increase of CD107a in the lin- NK cell population.

The situation in the rhCMV- animals looked different. Upon stimulation with Mamu-E- 721.221 cells, only 18.3% in the lin- NK cell population were CD107a+, the majority (64.8%) coming from the NKG2C-1/2- (4A8-Z199+) NK cell population and not the NKG2C-1/2+ (4A8+Z199+) population (24.2%) (**Figures 6A, B**). Upon Mamu-E trimer induction, only 15.3% of the lin- NK cells were CD107a+ with increased contribution of NKG2C-1/2+ (4A8+Z199+) cells raising from 24.2% to 45.5%. The contribution of the NKG2C-1/2- (4A8-Z199+) NK cell population decreased from 64.8% to 39.1%, suggesting NKG2A-mediated inhibition *via* binding of the induced Mamu-E (**Figures 6A, B**). These data show that the NK cell population in rhCMV- animals is dominated by 4A8- NK cells, i.e. NKG2A-expressing NK cells, that are inhibited by Mamu-E, whereas the NK cell population in rhCMV+ animals is dominated by 4A8+ NK cells, i.e. NKG2C-1/2-expressing NK cells, that are stimulated by Mamu-E.

Another fingerprint of NK cell stimulation is induction of IFN-gamma, which was measured in rhCMV+ donors upon stimulation with Mamu-E+ or with Mamu-E- 721.221 cells. While the incubation with Mamu-E- 721.221 cells increased the proportion of IFN-gamma expressing lin- NK cells from 8% to 48%, Mamu-E induction in the 721.221 target cells further increased IFN-gamma to 66% of the lin-NK cells (**Figure 6D**). This increase in IFN-gamma expression could markedly be attributed to the NKG2C-1/2+ (4A8+Z199+) NK cells (**Figure 6E**).

In summary, our functional analyses clearly demonstrated that the adaptive NK cells identified by antibody 4A8 induce strong degranulation and interferon-gamma expression upon recognition of Mamu-E by CD94/NKG2C-1/2 and that CMV infection increased the frequency of these degranulating adaptive NK cells.

#### Higher cell surface expression but similar degranulation of NKG2C-2 with different stalk lengths

**Figure 7:**
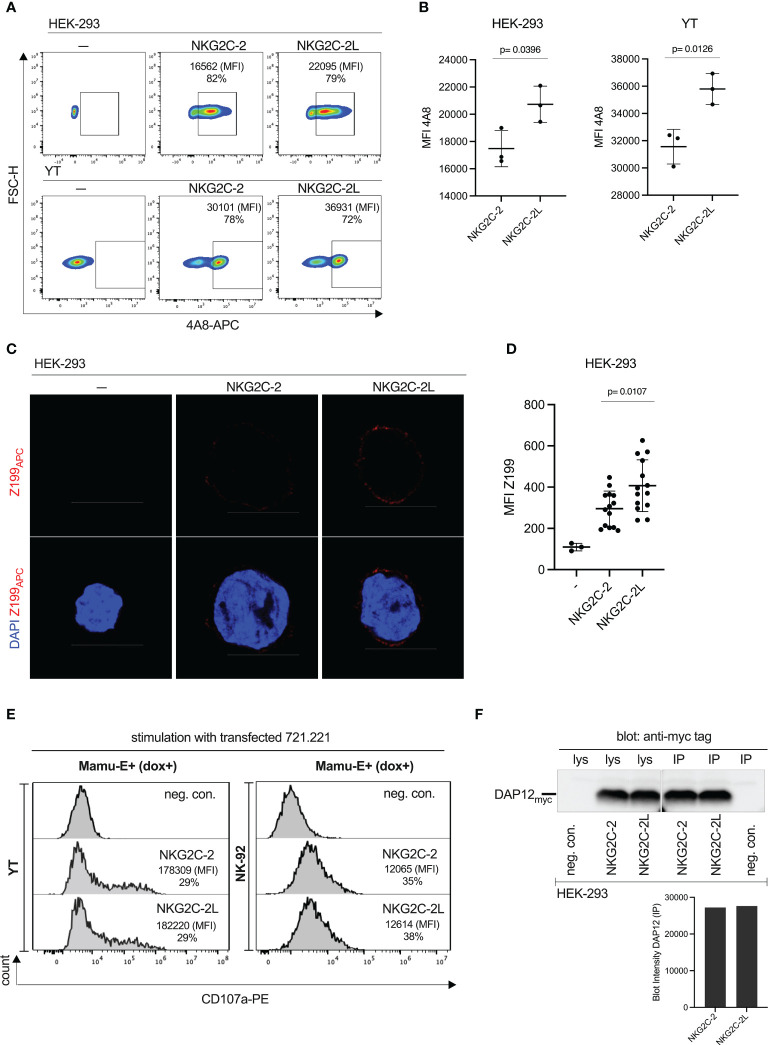
Functional consequences of stalk length polymorphism of CD94/NKG2C-2. **(A)** Flow cytometry analysis of stably transfected HEK-293 cells and YT cells expressing either NKG2C-2 with a regular stalk (encoded by exon 3B, see **Figure S7**) or NKG2C-2L with a longer stalk (encoded by exons 3A and 3B) with antibody 4A8. Percentages of 4A8+ cells and MFI are indicated. **(B)** MFI of antibody 4A8 in NKG2C-2 and NKG2C-2L transfected HEK-293 or YT cells. **(C)** HEK-293 cells either untransfected or transiently transfected with either NKG2C-2 or NKG2C-2L were stained with clone Z199 antibody and analyzed by confocal microscopy. Binding of Z199 is shown in red, cell nuclei stained with DAPI are shown in blue. Scale bar represents 10 µm. **(D)** Calculated MFI of 14 randomly chosen individual Z199+ cells expressing NKG2C-2 or NKG2C-2L in transfected HEK-293 cells. **(E)** Untransfected (-) or NKG2C-2 or NKG2C-2L transfected YT or NK-92 cells were incubated with 721.221 expressing dox-induced single-chain Mamu-E trimer. Percentage and MFI of CD107a+ YT and NK-92 cells are shown in the histograms. **(F)** Co-immunoprecipitation of myc-tagged DAP12 and short-stalk NKG2C-2 or long-stalk NKG2C-2L isotype. Immunoprecipitation was performed with antibody 4A8 as indicated (IP). Western blot analysis was performed with anti-myc antibody of cell lysates (lys) and immunoprecipitations from transfected HEK-293 cells expressing NKG2C-2 or NKG2C-2L (upper panel). Untransfected HEK-293 served as negative control (neg.con.). Densitometry of the DAP12 bands of IP experiments was performed and is shown in the lower panel.

During analysis of RT-PCR clones for the generation of expression constructs, we noticed that a considerable proportion of NKG2C-2 encoding cDNA clones contained a longer stalk-encoding region. The stalk is encoded by exon 3. Similar to previously published reports in human *NKG2C* and *NKG2E* (42) we also noted a duplicated genomic region between exon 2 and 4 in the three rhesus macaque *NKG2C* genes (**Figure S7**). While for *NKG2C-1* only exon 3A can be used due to a mutated splice acceptor at the downstream exon 3B (**Figure S7**), we found either exon 3A or exon 3A and 3B in *NKG2C-2* cDNA clones. A longer stalk region was also found by others (43, 44). Though not exhaustively searched for variants, we noticed only exon 3B in NKG2C-3 clones (**Figure S7**). In order to figure out whether this stalk elongation has functional consequences on expression strength and degranulation, we stably transfected the short and long stalk versions of NKG2C-2 together with rhCD94 and rhDAP12 in HEK-293 as well as in YT cells. We achieved similar levels of %-positive cells for both constructs in both cell lines when we used antibody 4A8 (**Figure 7A**). However, the MFI of the longer stalk version of CD94/NKG2C-2 was higher (p=0.0126, p=0.0396; **Figure 7B**). The same was found in confocal microscopy of transiently transfected HEK-293 cells where the stronger cell surface expression was evident with antibody Z199 (**Figures 7C, D**; p=0.0107). Yet, this higher cell surface expression of the long-stalk NKG2C-2 receptor did result in similar or only marginally higher degranulation in transfected YT and NK-92 cells upon stimulation with dox-induced Mamu-E trimer-expressing 721.221 cells (**Figure 7E**). In order to test whether there are differences in the two NKG2C-2 stalk isoforms in association with the DAP12 adaptor protein that might explain the latter findings, we performed respective co-immunoprecipitation experiments. We detected similar amounts of DAP12 in the Co-IPs (**Figure 7F**), indicating that DAP12 association is not different between the two NKG2C-2 stalk isoforms.

These results illustrated that the longer stalk augmented the expression of NKG2C-2, but this feature does not result in a clearly increased degranulation capacity.

## Discussion

C-type lectin-like receptors CD94/NKG2A and CD94/NKG2C play important roles in the regulation of NK cells as well as T cells (3–5, 45–51). This opens perspectives for treatment of certain cancers (3, 5, 46, 52) or in allogeneic transplantation of B2M-negative pluripotent stem cells expressing a single-chain HLA-E trimer (53). Hence, the CD94/NKG2A receptor is regarded as immune checkpoint. Indeed, repeated stimulation of NK cells induces expression of NKG2A (54, 55) that counteracts the stimulation-induced exhaustion of these cells (54, 56). Likewise, acute lymphoblastic leukemic cells downregulate HLA-E to much lower levels than healthy B cells from the same patient (57), but still these HLA-E levels are sufficient to ensure inhibition *via* CD94/NKG2A (58). Not surprisingly, this fine-tuned balance of HLA-E and CD94/NKG2A is exploited by HCMV upon downregulation of classical HLA class I and stabilization of HLA-E expression *via* the HCMV-encoded UL40 protein-derived VMAPRTL(I/V/F)L peptide (17, 59). Concomitantly, this stabilization induces an increase in CD94/NKG2C-expressing NK cells in many HCMV+ individuals as response to this infection (12, 13).

Infection of rhesus macaques with CMV is very similar to humans (60, 61), but detailed analyses of its impact on the CD94/NKG2 receptors on the protein level was hampered due to lack of specific anti-rhesus CD94 and NKG2 antibodies and that anti-human NKG2A antibody clone Z199 does not discriminate between CD94/NKG2A and CD94/NKG2C of rhesus macaques (35, 36). Moreover, we recently described an expansion of NKG2C-encoding genes in the rhesus macaque genome with presence of three *NKG2C* genes that were designated *NKG2C-1*, *NKG2C-2*, and *NKG2C-3* (19). As we were interested in the specific detection of NKG2C-expressing rhesus macaque NK cells, we immunized mice with NKG2C-2 and identified two antibody hybridoma clones showing identical specificity: in contrast to Z199 that reacts with all four rhesus macaque CD94/NKG2 proteins, our clones 4A8 and 7B1 react only with CD94/NKG2C-1 and CD94/NKG2C-2 heterodimers, but neither with CD94/NKG2A nor CD94/NKG2C-3. Using 4A8 in combination with Z199 we could identify in rhCMV-infected rhesus macaques a large population of 4A8+ NK cells expressing CD94/NKG2C-1 and/or CD94/NKG2C-2. In contrast, rhCMV-negative macaques exhibited fewer Z199+ NK cells and the fraction of NKG2C-1/2+ NK cells among this Z199+ population was much smaller as compared to rhCMV+ animals. Thus, rhCMV infection leads to an increase of NK cells that express NKG2C-1/2, a finding that is similar to human CMV infection where an expansion of NKG2C-expressing NK cells is frequently found.

These data on protein level confirms and extends previous reports by others who utilized RNA flow technique to detect transcription of a single *NKG2C* gene (62, 63). Interestingly, one of these two studies identified co-transcription of *NKG2A* and *NKG2C* in a large proportion of NK cells and that rhCMV infection increased the frequency of *NKG2C*+*NKG2A*- NK cells (62). To investigate this further, we used an arbitrarily chosen rhCMV+ animal of our colony in a small-scale single cell RNA seq experiment. This analysis demonstrated that the vast majority of 4A8+ NK cells transcribe in addition to *NKG2C-1* and *NKG2C-2* also the *NKG2C-3* and *NKG2A* genes (**Figure 3B**). This finding indicates that regulation of these genes might differ between rhesus macaques and human, where there is more or less exclusive expression observed for NKG2A and NKG2C under normal conditions in healthy blood donors (12). Yet, degranulation assays and IFN-gamma expression of this rhesus macaque NKG2C-1/2+ NK cell population clearly showed enhanced effector functions in *ex vivo* NK cells (**Figure 6**).

The simultaneous expression of NKG2A and NKG2C in human NK cells results in inhibition (64). The enhanced number of stimulatory NKG2C receptors in macaques might be one factor to explain the high effector function of macaque NKG2C-1/2+ NK cells despite NKG2A expression. A further factor is likely the higher avidity of both CD94/NKG2C-1 and CD94/NKG2C-2 for Mamu-E compared to CD94/NKG2A. Binding of HLA-E to NKG2 receptors is modulated by different peptides bound to Mamu-E (17, 65). The possibility that we had just selected a peptide by chance that elicits strong binding by NKG2C but not NKG2A receptors appears rather unlikely as we choose VMAPRTLLL as the binding peptide in the Mamu-E trimer. Firstly, this peptide is the most frequent one among the leader sequences of rhesus macaque Mamu-A, Mamu-B and Mamu-AG (the functional counterpart of HLA-G) allotypes (see IPD database), and secondly, it is contained within the rh67 protein encoded by the rhCMV genome and was recently shown to stabilize HLA-E expression (66). Therefore, our experimental setting with VMARTLLL-loaded Mamu-E is of biological significance and reflects the *in-vivo* situation of Mamu class I protein expression and infection with rhCMV. A further explanation for the co-expression of NKG2A and the three NKG2C receptors is chronic stimulation of these NK cells. Previous works by others showed induced NKG2A expression on NKG2C+ human NK cells after prolonged stimulation with IL-12 (54) or IL-15 (55) and that simultaneous signaling *via* induced NKG2A and *via* chronic engagement of stimulatory receptors counteracts exhaustion of NK cells that would otherwise be seen without induction and engagement of NKG2A (56). The question remains why these NKG2C-1/2+ NK cells are not autoreactive in these healthy rhesus macaques. Ongoing studies are currently performed to answer this question and to figure out the role of expression strength of Mamu-E in this context.

Our data clearly showed the increase of CD94/NKG2C-1 and/or CD94/NKG2C-2 expressing NK cells as a consequence of rhCMV infection. To analyze whether this subpopulation of 4A8+ NK cells display similar characteristics as HCMV-associated adaptive NK cells, we performed qRT-PCR experiments with sorted lin- NK cells from rhCMV+ and rhCMV- animals. Similar to human CD94/NKG2C+ adaptive NK cells, we found decreased transcription of *IL12RB2*, *ZBTB16* and *SH2D1B* and increased transcription of *IFNG* genes. Hence, there is evolutionary conservation of the molecular characteristics of adaptive NK cells in primates. In contrast to human adaptive NK cells that are further characterized by strongly decreased FCER1G expression (9, 18), we found slightly increased transcription of *FCER1G* in rhCMV+ rhesus macaques as compared to rhCMV- animals (**Figure 4**). The most probable reason for this difference between human and rhesus macaque adaptive NK cells is that macaques use FCER1G as adapter protein for their stimulatory KIR receptors and not DAP12 (20) as in human NK cells and, therefore, rely on continued expression of this adaptor protein. However, a report by Shah and colleagues describes a subpopulation of rhesus macaque NK cells that lacks expression of the *FCER1G*-encoded FCR-gamma adapter protein and expands in rhCMV-infected rhesus macaques (67). These contradictory data of FCR-gamma are interesting and may indicate a certain heterogeneity in the characteristics of CMV-associated adaptive NK cells in rhesus macaques, similar to what is known for SYK and SH2D1B in human adaptive NK cells which differ in the level of downregulation between individuals ( (9) and own unpublished observations).

Among the three NKG2C proteins, NKG2C-2 is of particular biological significance due to its prominent gene transcription (**Figures 3A, D**) and its high binding avidity to Mamu-E (**Figure 5B**). A further peculiarity of NKG2C-2, which is not found in human NKG2C, is a polymorphism of its stalk length. Though not tested systematically, a considerable proportion of our cloned NKG2C-2 cDNA sequences show alternative splice products that contained two stalk-encoding exons. We were interested in figuring out whether this stalk polymorphism has obvious functional consequences. In comparison with the regular stalk length version of NKG2C-2, we noticed higher cell surface expression for the longer stalk version (**Figures 7A-D**). Though, this stronger expression was not mirrored in stronger degranulation upon binding of the Mamu-E ligand (**Figure 7E**). Possible explanations for these observations are that **1)** both antibodies 4A8 and Z199 have better access to their epitopes in longer-stalk NKG2C-2 and due to more efficient binding the cell surface expression appears higher but the similar number of receptors is reflected by similar degranulation, or **2)** the longer-stalk NKG2C-2 is present in higher numbers on the cell surface but the signaling capacity is lower than in the regular stalk version. We tested the latter hypothesis by co-immunoprecipitation of the DAP12 adapter protein but did not find differing amounts of associated DAP12 for the shorter and longer stalk NKG2C-2 isotypes (**Figure 7F**). Future studies have to figure out the functional role of the stalk polymorphism. Interestingly, the stalk region of inhibitory Ly49C and stimulatory Ly49H receptors in the mouse are the recognition structure of the MHC class I-like m157 protein of mouse CMV (68).

The development and usage of monoclonal antibodies specifically recognizing rhesus macaque stimulatory NKG2C proteins significantly enhanced the knowledge of consequences of rhCMV infection on NK cells on the protein level. In combination with anti-human NKG2A antibody Z199, our monoclonal anti-rhesus macaque NKG2C-1/2 antibodies offer excellent possibilities not only for regular multicolor flow cytometry analysis but for sorting of live cells and subsequent functional studies of adaptive NK cells in this biomedically important nonhuman primate model of human infectious diseases. Future studies should also strive for establishment of specific monoclonal anti-rhesus macaque NKG2A antibodies.

## Data availability statement

The datasets presented in this study can be found in online repositories. The names of the repository/repositories and accession number(s) can be found below: GEO accession GSE211613.

## Ethics statement

Ethical review and approval was not required for obtaining the samples described in this study because rhesus macaques (*Macaca mulatta*) kept at the German Primate Center under human care are undergoing a yearly routine veterinary health control that includes sampling of peripheral blood. Small aliquots of these samples were obtained from the Animal Husbandry Unit for diagnostic phenotyping. Spleen samples from autopsies of euthanized animals were obtained from the Pathology Unit. Permission for the immunization of mice was obtained from the responsible authority, the Lower Saxony State Office for Consumer Protection and Food Safety, permit number 33.42502-05-A-029/09.

## Author contributions

LW conceived and designed the study, analyzed data, and wrote the manuscript. MZH contributed to experiment design and performed experiments, analyzed data and prepared figures, and wrote sections of the manuscript. CH performed bioinformatic analyses and prepared figures. BP established monoclonal antibodies. AS and KM-R contributed samples. AK performed CMV screening of rhesus macaques. GS performed single cell RNA sequencing. RD performed mouse immunizations. All authors contributed to manuscript revision, read, and approved the submitted version.

## Funding

This study was supported by grants from the German Research Foundation (grant WA 1033/5-1) and the German Center for Cardiovascular Research (grant 81X2300204) to LW and by institutional funds of the German Primate Center, Leibniz Institute for Primate Research, which also covered the open access publication fee.

## Acknowledgments

The authors would like to thank Markus Uhrberg for helpful comments and Ellen Eckermann-Felkl, Nico Westphal, Swantje Andreß, and Fabian Ludewig for expert technical assistance.

## Conflict of interest

The authors declare that the research was conducted in the absence of any commercial or financial relationships that could be construed as a potential conflict of interest.

## Publisher’s note

All claims expressed in this article are solely those of the authors and do not necessarily represent those of their affiliated organizations, or those of the publisher, the editors and the reviewers. Any product that may be evaluated in this article, or claim that may be made by its manufacturer, is not guaranteed or endorsed by the publisher.

## Supplementary material

Supplementary Figure 1 Schematic presentation of the different constructs used to express CD94/NKG2 receptors on the cell surface, or the extracellular part of NKG2C-2 or single-chain Mamu-E trimer as human IgG-Fc fusion protein, or doxycycline-induced expression of single-chain Mamu-E trimer on the cell surface.

Supplementary Figure 2 Quality control data for the single cell RNA seq analysis. **(A)** Quality control metrics for the 4A8 antibody positive NK cells. “percent.mt” indicates percentage of mitochondrial reads, “percent.ribo” ribosomal read content, “nCount_RNA” the number of reads, and “nFeature_RNA” the number of features per cell. All quality control metrics were calculated in R using Seurat and the custom *Macaca mulatta* genome annotation. Ribosomal genes were defined as genes with the biotype tag “rRNA”, mitochondrial genes were defined as genes with the prefix “MT” in their seqid. **(B)** PCA plot of sequenced single cells. Cells are colored regarding their cell cycle stage as calculated using Seurat’s function “CellCycleScoring” and Seurat’s “cc.genes.updated.2019” as cell cycle marker genes. Cell cycles are indicated by different color.

Supplementary Figure 3 Complete gating strategy for the flow cytometry analysis of PMBCs of a rhCMV+ rhesus macaque using lineage marker antibodies (CD3CD14CD20), and anti-human NKG2A antibody Z199, anti-human NKG2C antibodies 134522 and 134591, as well as anti-rhesus macaque NKG2C-1/2 antibody 4A8, respectively. Gating strategies follows: lymphocytes, singlets, viable cells (zombie aqua BV510-negative), lin- cells (= NK cells), Z199+ or 4A8+ or 134522+ or 134591+ cells.

Supplementary Figure 4 Complete gating strategy for the flow cytometry analysis of PMBCs of a rhCMV- and a rhCMV+ rhesus macaque using lineage marker antibodies (CD3CD14CD20), anti-rhesus macaque pan-KIR3D antibody 1C7 (22), and anti-rhesus macaque NKG2C-1/2 antibody 4A8. Gating strategies follows: lymphocytes, singlets, viable cells (zombie aqua BV510-negative), lin- cells (= NK cells), 1C7+ NK cells, 4A8+ cells among the 1C7+ NK cells.

Supplementary Figure 5 Characterization of the specificity of primers for the detection of rhesus macaque *NKG2A*, *NKG2C-1*, *NKG2C-2* and *NKG2C-3* gene transcripts by qRT-PCR. Stably transfected HEK-293 cells expressing the different *NKG2* genes are indicated on the x-axis. Mean cycle threshold (Ct) values of triplicate measurements of a single experiment are indicated.

Supplementary Figure 6 **(A)** Complete gating strategy for the flow cytometry analysis of untreated or IL-2/IL-15-treated PMBCs of a rhCMV+ rhesus macaque using lineage marker antibodies (CD3CD14CD20), and anti-rhesus macaque NKG2C-1/2 antibody 4A8. Gating strategies follows: lymphocytes, singlets, viable cells (zombie aqua BV510-negative), lin- cells (= NK cells), 4A8+ cells. The percentages of lin-4A8+ NK cells of a rhCMV+ rhesus macaque are shown for untreated cells and for cells stimulated overnight with IL-2 and IL-15 (below the gating strategies). **(B)** Example of complete gating strategy for the flow cytometry analysis of degranulation assays. PBMCs of a rhCMV+ rhesus macaque stained with lineage markers, anti-CD107a, and antibody clones 4A8 and Z199. Lin- NK cells were analyzed for CD107a, and these CD107a+ NK cells were then analyzed for binding of 4A8 and Z199. Gating strategies follows: lymphocytes, singlets, viable cells (zombie aqua BV510-negative), lin- cells (= NK cells), CD107a+ cells, 4A8+Z199+ and 4A8-Z199+ cells.

Supplementary Figure 7 Genomic organization of *NKG2C-1*, *NKG2C-2* and *NKG2C-3* exon 2 to 4 sequences and variation of stalk regions encoded by exons 3A and 3B. For exon 2 and exon 4 only the 3’ end and the 5’ part are shown, respectively.
