# Supplementary material for: Detailed phenotypic and functional characterization of CMV-associated adaptive NK cells in rhesus macaques
Source: Front Immunol. 2022 Nov 25;13:1028788. doi: 10.3389/fimmu.2022.1028788 (PMC9742600; doi:10.3389/fimmu.2022.1028788)

Figure S1

constructs for cellular expression of CD94/NKG2 receptors:

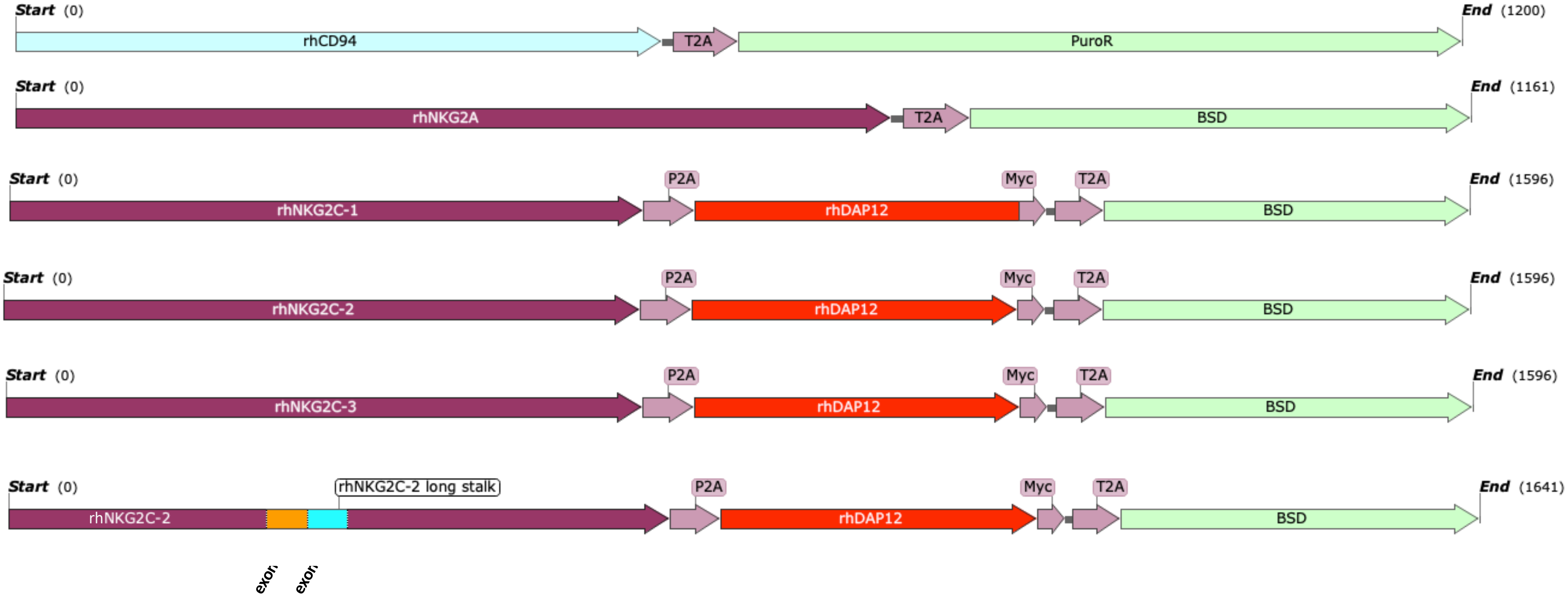

constructs for expression of hIgG1-Fc fusion proteins:

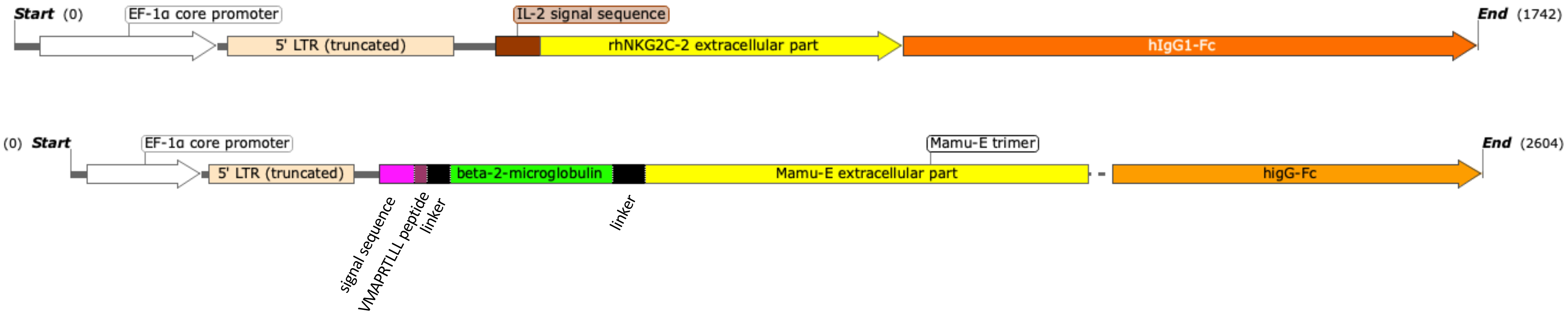

construct for doxycyclin-induced expression of a single-chain Mamu-E trimer on the cell surface:

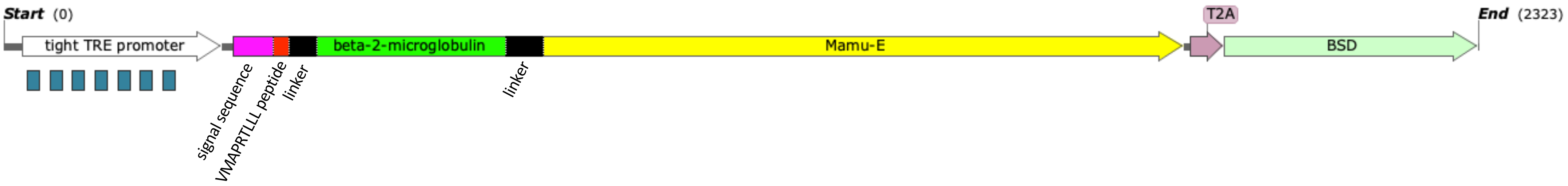

Supplement: Supplementary Figure 1 — Schematic presentation of the different constructs used to express CD94/NKG2 receptors on the cell surface, or the extracellular part of NKG2C-2 or single-chain Mamu-E trimer as human IgG-Fc fusion protein, or doxycycline-induced expression of single-chain Mamu-E trimer on the cell surface. [file DataSheet_1.pdf]
