# Supplementary figures and images for: Detailed phenotypic and functional characterization of CMV-associated adaptive NK cells in rhesus macaques
Source: Front Immunol. 2022 Nov 25;13:1028788. doi: 10.3389/fimmu.2022.1028788 (PMC9742600; doi:10.3389/fimmu.2022.1028788)

Figure S2

**A**

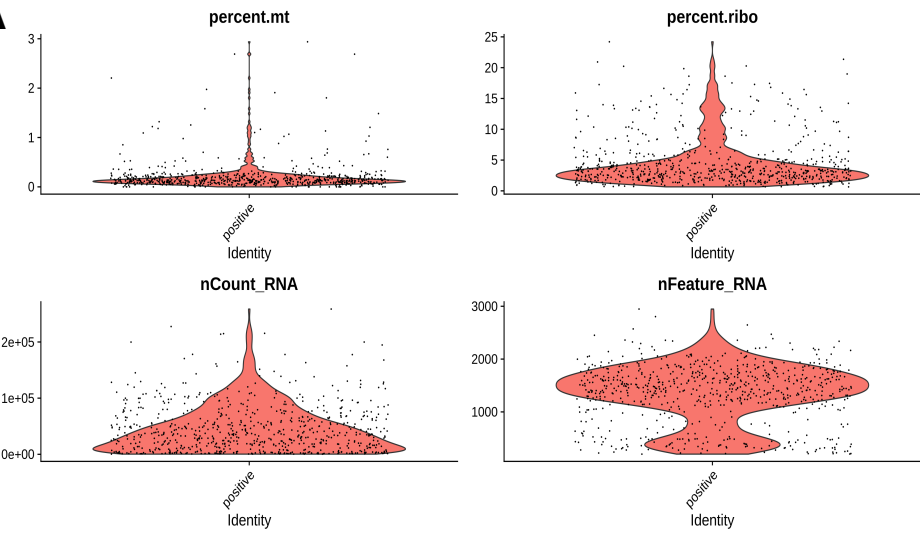

**B**

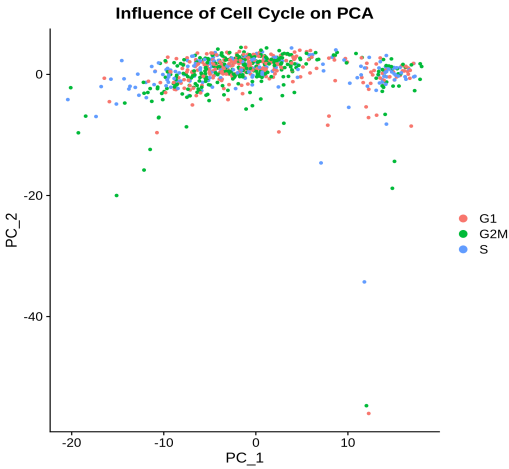

Supplement: Supplementary Figure 2 — Quality control data for the single cell RNA seq analysis. (A) Quality control metrics for the 4A8 antibody positive NK cells. “percent.mt” indicates percentage of mitochondrial reads, “percent.ribo” ribosomal read content, “nCount_RNA” the number of reads, and “nFeature_RNA” the number of features per cell. All quality control metrics were calculated in R using Seurat and the custom Macaca mulatta genome annotation. Ribosomal genes were defined as genes with the biotype tag “rRNA”, mitochondrial genes were defined as genes with the prefix “MT” in their seqid. (B) PCA plot of sequenced single cells. Cells are colored regarding their cell cycle stage as calculated using Seurat’s function “CellCycleScoring” and Seurat’s “cc.genes.updated.2019” as cell cycle marker genes. Cell cycles are indicated by different color. [file DataSheet_2.pdf]

**Figure S3**

rhPBMCs (rhCMV+)

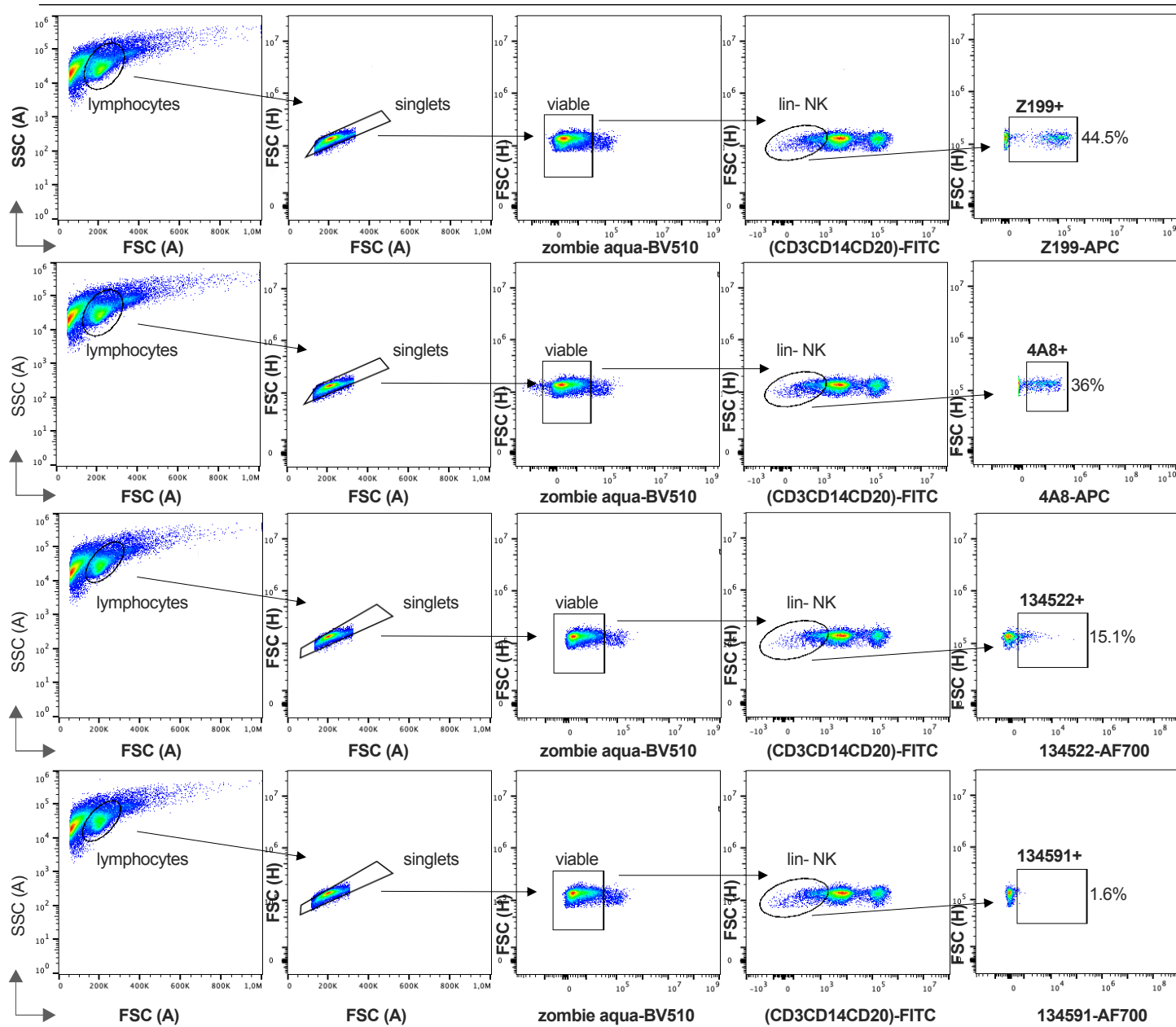

Supplement: Supplementary Figure 3 — Complete gating strategy for the flow cytometry analysis of PMBCs of a rhCMV+ rhesus macaque using lineage marker antibodies (CD3CD14CD20), and anti-human NKG2A antibody Z199, anti-human NKG2C antibodies 134522 and 134591, as well as anti-rhesus macaque NKG2C-1/2 antibody 4A8, respectively. Gating strategies follows: lymphocytes, singlets, viable cells (zombie aqua BV510-negative), lin- cells (= NK cells), Z199+ or 4A8+ or 134522+ or 134591+ cells. [file DataSheet_3.pdf]

Figure S4

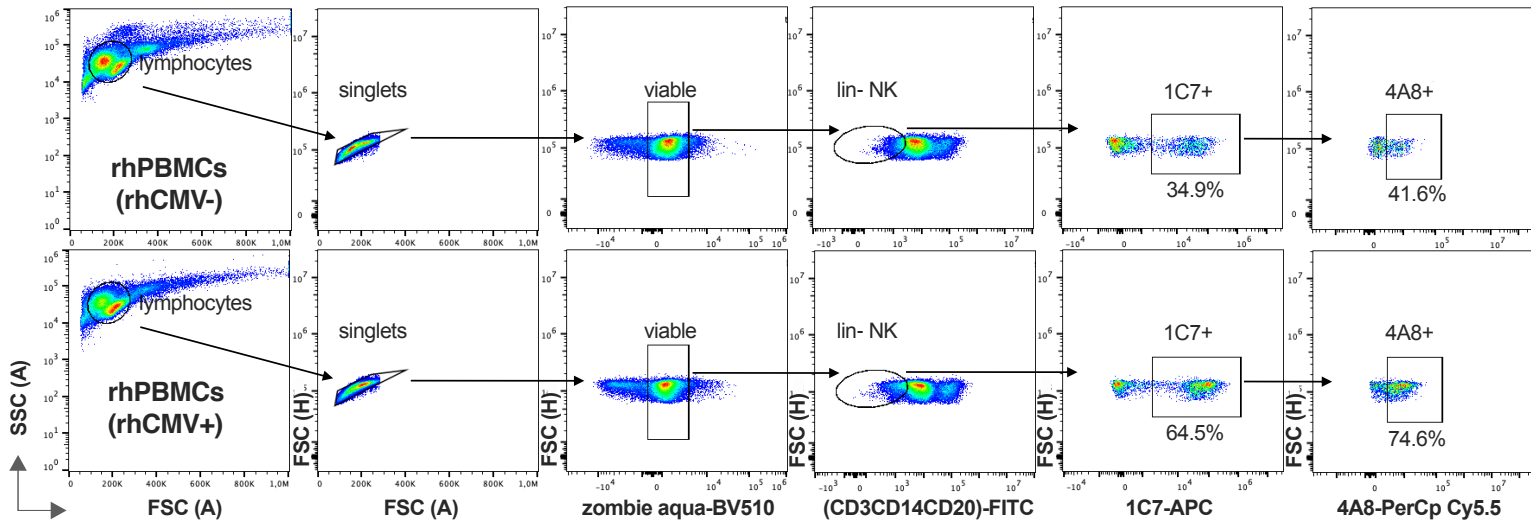

Supplement: Supplementary Figure 4 — Complete gating strategy for the flow cytometry analysis of PMBCs of a rhCMV- and a rhCMV+ rhesus macaque using lineage marker antibodies (CD3CD14CD20), anti-rhesus macaque pan-KIR3D antibody 1C7 (22), and anti-rhesus macaque NKG2C-1/2 antibody 4A8. Gating strategies follows: lymphocytes, singlets, viable cells (zombie aqua BV510-negative), lin- cells (= NK cells), 1C7+ NK cells, 4A8+ cells among the 1C7+ NK cells. [file DataSheet_4.pdf]

Figure S5

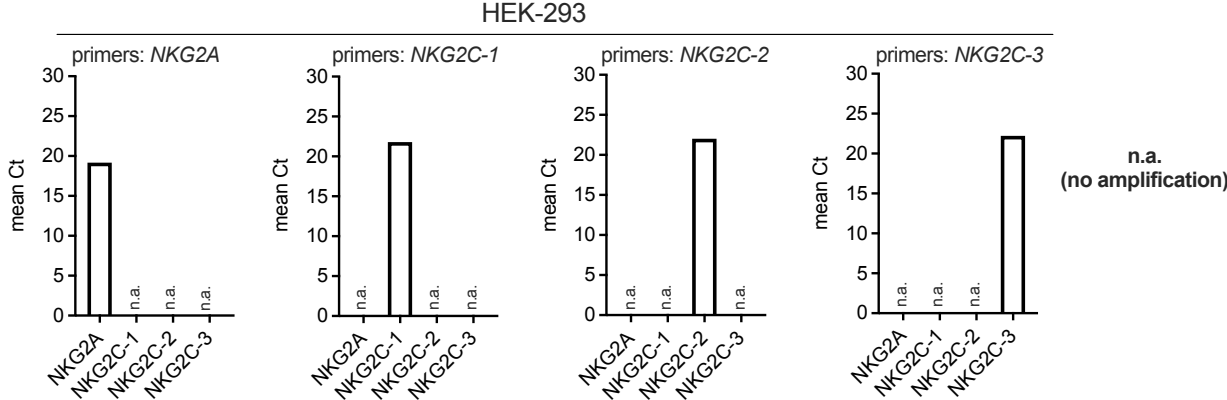

Supplement: Supplementary Figure 5 — Characterization of the specificity of primers for the detection of rhesus macaque NKG2A, NKG2C-1, NKG2C-2 and NKG2C-3 gene transcripts by qRT-PCR. Stably transfected HEK-293 cells expressing the different NKG2 genes are indicated on the x-axis. Mean cycle threshold (Ct) values of triplicate measurements of a single experiment are indicated. [file DataSheet_5.pdf]

**Figure S6****A**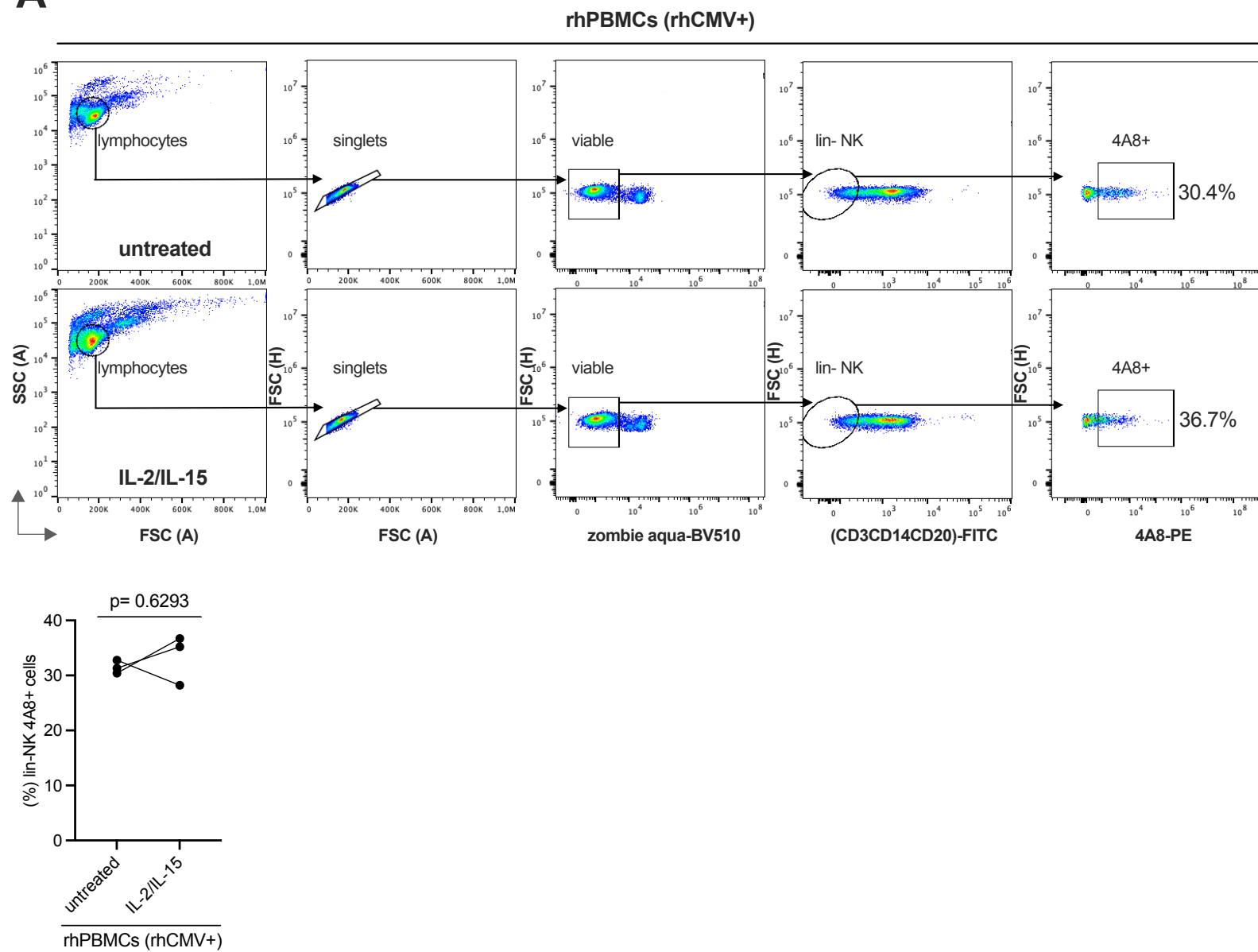**B**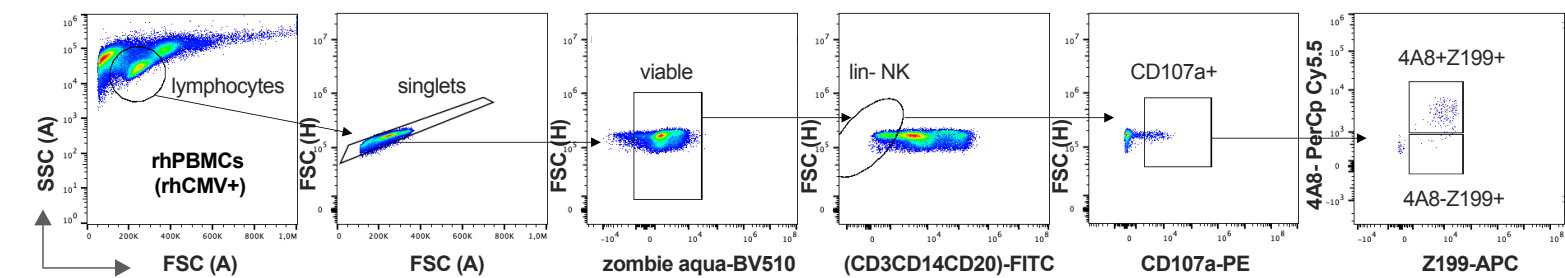

Supplement: Supplementary Figure 6 — (A) Complete gating strategy for the flow cytometry analysis of untreated or IL-2/IL-15-treated PMBCs of a rhCMV+ rhesus macaque using lineage marker antibodies (CD3CD14CD20), and anti-rhesus macaque NKG2C-1/2 antibody 4A8. Gating strategies follows: lymphocytes, singlets, viable cells (zombie aqua BV510-negative), lin- cells (= NK cells), 4A8+ cells. The percentages of lin-4A8+ NK cells of a rhCMV+ rhesus macaque are shown for untreated cells and for cells stimulated overnight with IL-2 and IL-15 (below the gating strategies). (B) Example of complete gating strategy for the flow cytometry analysis of degranulation assays. PBMCs of a rhCMV+ rhesus macaque stained with lineage markers, anti-CD107a, and antibody clones 4A8 and Z199. Lin- NK cells were analyzed for CD107a, and these CD107a+ NK cells were then analyzed for binding of 4A8 and Z199. Gating strategies follows: lymphocytes, singlets, viable cells (zombie aqua BV510-negative), lin- cells (= NK cells), CD107a+ cells, 4A8+Z199+ and 4A8-Z199+ cells. [file DataSheet_6.pdf]
