# Supplementary material for: Detailed phenotypic and functional characterization of CMV-associated adaptive NK cells in rhesus macaques
Source: Front Immunol. 2022 Nov 25;13:1028788. doi: 10.3389/fimmu.2022.1028788 (PMC9742600; doi:10.3389/fimmu.2022.1028788)
Supplement: Supplementary Figure 7 — Genomic organization of NKG2C-1, NKG2C-2 and NKG2C-3 exon 2 to 4 sequences and variation of stalk regions encoded by exons 3A and 3B. For exon 2 and exon 4 only the 3’ end and the 5’ part are shown, respectively. [file DataSheet_7.pdf]

**Figure S7**

exon 2, exon 3A, exon 3B, exon 4, *duplicated region*, ***splice signals***

```
>NKG2C-1:         IVLMATVLKTIVLIPLLEQNNSSPNTTTQK---------------VRHCGHCPEE
>NKG2C-2 short:   IVLMATVLKTIVLIPFPEQSNSSLNTRTQK---------------VRHCGHCPEE
>NKG2C-2 long:    IVLMATVLKTIVLIPFPEQSNSSLNTRTQKVLEQNNSFPNTRTQKVRHCGHCPEE
>NKG2C-3:         IVLMATVLKTVVLIP---------------FLEQNNSFPNTRTQKVRHCGHCPEE
```

>*NKG2C-1* in BAC CH250-201L20 (GenBank AC201868.4) sequence:

```
          attgtc ctgatggcca
    79441 ctgtgttaaa aacaatagtt cttattcctc gtaagcgtat ttttgaaaga ttagacggga
    79501 aagttttact ttaatgcttg gaagtgcctc aaaatatttc atactgttga agaatagaac
    79561 tctcattgta gtgtttattt cagagatcta ttacttcatt tatttttata gaaaaagtta
    79621 atttcattaa agattgtccc cattttaaat aacatacaaa gtttcaaagt aagaaactaa
    79681 acccattatg gtttatctag atattagttt ttacgaaaat cattttaatt tttctgttac
    79741 agtcctggag cagaacaatt cttccccgaa tacaacaacc cagaaaggta cattttttatt
    79801 ttcaacgttc agatattagt ataatttgga cccaaaagtg atatggttat tctgaacttt
    79861 tcacaacaca aataacaaaa tcattgtaga gaatatgtgt ttttttgtgt gtttgtaatc
    79921 tatctaagta tctatctatc tatctatcta tctatctatc tatctatcta tacacataca
    79981 cacacaatgt attttctgat ttcataattc aaaggcatac tataggagaa aagaatttag
    80041 aaaaacaaat taatttttga aagtggttac atcaaatact ataagcgatg gtgaagtttt
    80101 gtgctaaagt ctttaaaaat actttttca aagatttatt catttatttt tataggaaac
    80161 gttaatttta ttaaagattg ttcccatttt aaataacaca caaagtttca aagtaagaaa
    80221 ctaaattcat tatggtttat ctagatacta gttttataa aactcatttt aatttttcta
    80281 ttacaatcct ggagcagaac aattctttcc cgaatacaag aacccagaaa ggtacacttt
    80341 tattttcatc attcagatat tagtacaatt tatatttgt gtctgtttta aggcatgtaa
    80401 aagaatgatg gcatttttgc agaaaataag ccataaaatt cagccataaa tatttataaa
    80461 caaagattat aaggcagcat ttccttttct ccgataagta gaaatactca cttaaaatca
    80521 ttctaccctc tttctcccaa ttaacagaag tttcctactg ctatgagatg atatgaaatg
    80581 aataatttta ctatactaaa aaagcagttg tgtatcagtg acgttcaaga catgtgtaga
    80641 gtgtattttt gtttgttggt ttgctttata tggaaacaca attggtgatg agaggctagc
    80701 ccttgtctgt gcatgtgtgt atgactgact cggttattaa aaaatatata ttcataagcc
    80761 tgtaaggatg cataaatatg ttaagcacat ataggtttat actgttggaa atacgtaaac
    80821 taattttcat ttgtaaacat tcatattgtt ctgcatagta attcatatct ttatttagta
    80881 cgtcattgtg gccattgtcc tgaggag
```

>*NKG2C-2* in BAC CH250-201L20 sequence:

```
          attgtc ctgatggcca ctgtgttaaa aacaatagtt cttattcctt gtaagcacat
    95161 tcttgaaaga ttagacggga acattttact ttaatgcttg gaagtgcctc aaaatatttc
    95221 atactgttga agaatagaac tctcattgta gtgtttattt cagagatcta ttacttcatt
    95281 tattttttac agaaaaagtt aatttcatta aagattgtcc ccattttaaa taacacacaa
    95341 agtttcaaag taagaaacta aacttgttat ggtttatctg gatattagtt tttacaaaaa
    95401 tcattttaat ttttctatta cagtcccgga gcagagcaat tcttccctga atacaagaac
    95461 ccagaaaggt acatttttat tttcaacgtt cagatattag tataatttgg acccagaagt
    95521 aatatggtta ttctgaactt ttcacaacat aaataacaaa atcattgtag agaatatgtg
    95581 tttatttttg tgtgtgtgta acctatctat atatatatat acacacacat acacatacga
    95641 tgtattttct gaattcataa ttaaaatgca tgctatagga gaaaagtatt tagaaaaacg
    95701 aattaatttt tgaaagtggt tatgtcaaat actacaagag atggtgaagt ttagtgctaa
    95761 agtctttaaa aatatttctt tcgaagatct attcatttat ttttatagaa aaagtgaatt
    95821 ttattaaaga ttgtccccat tttaaataac acacaaagtt tcaaagtaag aaactaaatt
    95881 cattatggtt tatctagata ttagttttta taaaactcat tttaattctt ctattacagt
    95941 cctggagcag aacaattctt tcccgaatac aagaacccag aaaggtacat atttatttta
    96001 aatgttctga tattagtaca atttatattt tgtgtctggt ttaaggcatg taaaagaatg
    96061 atggcatttt tgcagaaaat aagccataaa attcagccat aaatatttat aaacaaagat
    96121 tataaggcag catttccttt tctccgataa gtagaaatac tcaattaaaa tcattctacc
    96181 ctctttctcc caattaacag aagtttccta ctgctatgag atgatatgaa atgaataatt
    96241 ttactatcct aaaaaagcag ttgtgtatca gtgatgttca agacatgtgg agagtgtatt
    96301 tttgtttgtt ggtttgcttt atatgggaac acgattaggg atgacaggct aacccttgtc
    96361 tgtgcatgtg tgtatgactg actcggttat taaaaaatat atatttataa gccggtaagg
    96421 atgcataaat atgttaagca catataggtt tatactgttg caaatatgta aacgaatttt
    96481 catttgtaaa cattcatatt gttctgcata gtaattcata tctttattta gtacgtcact
    96541 gtggccattg tcctgaggag
```

>*NKG2C-3* in BAC CH250-201L20 sequence:

attgt cctgatggcc actgtgttaa aaacagtggt tcttattcct

110821 t**gt**aagcaca ttcttgaaag actagatggg aacattttac tttaatgctt ggaagtgcct

110881 cacaatattt catactgttg aagaatagaa ctctcattgt aacg*ttaatt tcagagatct*

*110941 attacttcat ttatttttat agaaaaagtt aatttcatta aagattgtcc ccattttaaa*

*111001 taacacacaa agtttcaaag taagaaacta aacttgttat ggtttatcta ggtattagta*

*111061 cataaaccat agcagagcaa ttctccggt****a*** ***g****ataaaccgg agcagagcaa ttcttccccg*

*111121 aatacaagaa cccagaaag****g*** ***t****acattttta ttttcaacat tcagatatta gtataatttg*

111181 gacccaaaag taatatggtt attctgaatt tttcacaaca taaataacaa aatcattgta

111241 gagaatatgt gtttgttttt tgtgtgtatg taatctatat atctatatat acacacacat

111301 acacatacaa cgtattttct gaattcaaaa ttcaaatgca tgctatagga gaaaagtatt

111361 tagaaaaaca aattaatttt tgaaagtggt tacgtcaaat actacaagag atggtgaagt

111421 tttgtgctaa agtctttaaa aata*tttctt tcgaagatct agtcatttat ttttatagaa*

*111481 aaagtgaatt ttattaaaga ctgtccccaa tttaaataac acacaaagtt tcaaagtaag*

*111541 aaactaaact cattatggtt tatctagata ttagttttta taaaactcat tttaattttt*

*111601 ctattac****ag****t cctggagcag aacaattctt tcccgaatac aagaacccag aaag****gt****acat*

*111661 atttatttta aatgttctga tagtacaatt tatatttt*gt gtctggttta aggcatgtaa

111721 aagaatgatg gcatttttgc agaaaataag ccataaaatt cagccataaa tatttataaa

111781 caaagattat aaggcggcat ttccttttct ccaataagta gaaatgctca cttaaaatca

111841 ttctaccctc tttctcccaa ttaacagaag tttcctactg ctatgagatg atatgaaatg

111901 aataatttta ctatcctaaa aaagcagttg tgtgtcagtg atgttcaaga catgtggaga

111961 gtgtattttt gtttgttggt ttgctttata tgggaacaca gttagggatg agaggctaac

112021 ccttgtctgt gcatgtgtgt atgactgact cggttattaa aaacatatat ttataagcct

112081 gtaaggatgc ataaatatgt taagcacata caggtttata ctgttgcaaa tatgtaaact

112141 aattttcatc tgtaaacatt catattgttc tgcacagtaa ttcatatctt tattt**ag**tac

112201 gtcactgtgg ccattgtcct gaggag
