# Supplementary material for: Detailed phenotypic and functional characterization of CMV-associated adaptive NK cells in rhesus macaques
Source: Front Immunol. 2022 Nov 25;13:1028788. doi: 10.3389/fimmu.2022.1028788 (PMC9742600; doi:10.3389/fimmu.2022.1028788)
Supplement: Supplementary file 8 [file Table_1.pdf]

Table S1 List of primers for comparative qPCR

Gene symbol	Forward primer	Reverse primer
rhRPL13A	CCTGGAGGAGAAGAGGAAAGAGA	TTGAGGACCTCTGTGTATTTGTCAA
rhNKG2A	CAATAGTTGTTATTCCCTCTAC	AGTTCTTTTCTCCTTACCAATAT
rhNKG2C-1	ATAGTTCTTATTCCTCTCCTG	AGACTAGAGTTCTTTGAAGCA
rhNKG2C-2	GACTGCCATTTTACCTTCCT	TACTGTAGATTCACACTGGTTT
rhNKG2C-3	TTTCACCATCCACATGGACT	GACTGATTTAAGTCTATCTAGA
rhSH2D1B	AGACTGTGAGACCTTGCTGC	GAGACACAGAGGCACAGGAC
rhIL12RB2	AGAGGACAAGACACCCACCT	ATTGACAGCAGTAACCTTGGC
rhZBTB16	TGCGGCTGAGAATGCACTTA	ACACAGCAGACAGAAGACGG
rhIFNG	ACTGCCAGGACCCATATGTAA	GTTCCATTATCTGCTACATCTGGA
rhFCER1G	TTTGGTTGAACAAGCAGCGG	TGCCTTTCGCACTTGGATCT
